# Dual-Ion Co-Storage via Solvation Structure Tuning Toward Ultrafast and Durable Zinc-Organic Batteries

**DOI:** 10.1007/s40820-026-02304-7

**Published:** 2026-07-28

**Authors:** Si Liu, Zhifeng Lin, Yanxia Yu, Haozhe Zhang, Xihong Lu

**Affiliations:** 1 School of Electronic and Information Engineering, School of Environmental and Chemical Engineering, Foshan University, Foshan, 528000 People’s Republic of China; 2 The Key Lab of Low-Carbon Chem & Energy Conservation of Guangdong Province, School of Chemistry, Sun Yat-Sen University, Guangzhou, 510275 People’s Republic of China; 3 School of Applied Physics and Materials, Wuyi University, Jiangmen, 529020 People’s Republic of China; 4 Pritzker School of Molecular Engineering, University of Chicago, Chicago, IL 60637 USA

**Keywords:** Zinc-organic batteries, Co-solute, Solvation structure, PTCDA

## Abstract

Aqueous Zn-organic batteries are promising due to the sustainability and tunability of organic cathodes. However, the critical challenge in their practical application lies in dissolution, degradation, and sluggish kinetics, ultimately degrading the cycling stability and rate capability. Herein, we demonstrate a simple effective electrolyte engineering strategy by introducing potassium chloride (KCl) as a co-solute into the ZnCl_2_ electrolyte to achieve high-performance Zn batteries utilizing 3,4,9,10-perylenetetracarboxylic dianhydride (PTCDA) as the cathode. Experimental studies and molecular dynamics simulations reveal that KCl addition not only enhances electrolyte conductivity but also modulates Zn^2+^ solvation environment to form [Zn(H_2_O)_2_Cl_4_]^2−^, significantly improving ion diffusion kinetics. Consequently, the Zn//PTCDA in the ZnCl_2_-KCl electrolyte exhibits a remarkable capacity of 124.7 mAh g^−1^ with an average voltage of 0.65 V, exceptional rate performance (56% capacity retention at 30 A g^−1^), and prolonged cycling performance (90.9% retention after 10,000 cycles). Experimental and density functional theory mechanistic studies unveil a new reversible Zn^2+^/K^+^ co-storage mechanism in the PTCDA cathode, where K^+^ acts as a charge shield and structural pillar, synergistically decreasing the ion migration energy barrier, enhancing reaction kinetics, and stabilizing the cathode structure. This work elucidates dual-ion storage chemistry and highlights rational electrolyte design for durable, high-power metal–organic batteries.

**Supplementary Information:**

The online version contains supplementary material available at 10.1007/s40820-026-02304-7.

## Highlights

- Introducing KCl into ZnCl_2_ electrolyte induces salting-out and suppresses 3,4,9,10-perylenetetracarboxylic dianhydride (PTCDA) dissolution.
- The reversible Zn^2+^/K^+^ co-storage mechanism of the PTCDA cathode was unveil.
- The assembled Zn//PTCDA battery exhibits superior electrochemical performance.
- A high capacity of 124.7 mAh g^−1^ (91.7% of the theoretical capacity) at 1 A g^−1^ is achieved.

**Supplementary Information:**

The online version contains supplementary material available at 10.1007/s40820-026-02304-7.

## Graphical abstract

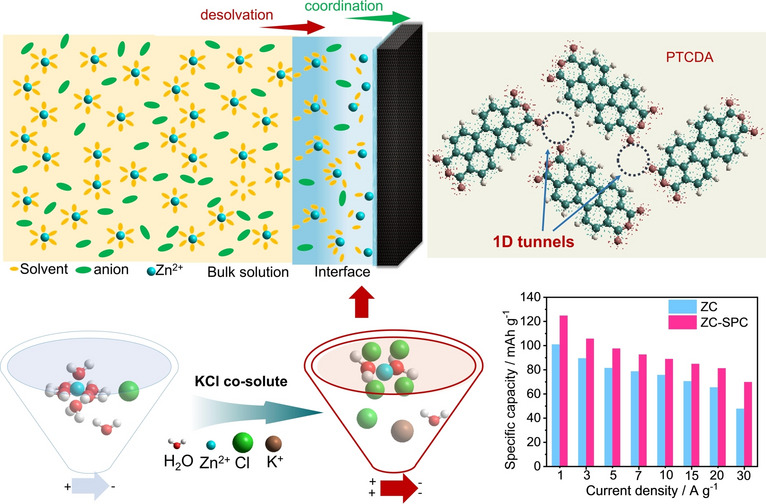

## Introduction

To satisfy the requirements of renewable energy storage and daily rapid charging, there is a critical requirement for energy storage devices that simultaneously deliver superior rate capability and high-power density [1, 2]. Owing to excellent energy density, lithium-ion batteries (LIBs) currently dominate the market for general-purpose energy storage [3, 4]. However, their organic electrolytes exhibit some intrinsic limitations, such as low ionic conductivity (~ 10^−3^ S cm^−1^) and flammability, leading to inadequate rate performance and safety hazards under rapid charging conditions. These issues have spurred the exploration of safer and more efficient alternatives. As a promising complement to diversify the energy storage landscape, aqueous metal ion batteries (such as Ca^2+^, Mg^2+^, Zn^2+^, Al^3+^) employ water-based electrolytes, which inherently eliminate fire hazards and overcome rate limitations arising from their inherent flame retardancy and superior ion transport capability (~ 1 S cm^−1^) [5, 6]. In particular, aqueous zinc ion batteries (AZIBs) are highly intriguing due to the compelling attributes of the Zn anode, including its remarkable theoretical capacity (820 mAh g^−1^, 5851 mAh cm^−3^), favorable redox potential (− 0.76 V vs. SHE), nontoxicity, cost-effectiveness, and easy processing [7–10]. Despite these attractive features, the practical commercialization of AZIBs is significantly hindered by the inadequate performance of typical cathode materials, particularly with respect to cycling stability, rate capability, and output power density. Substantial research has focused on designing high-performance cathode hosts, including manganese, vanadium, and molybdenum oxides and sulfides [11–16], Prussian blue analogs [17, 18], and organic compounds [19–21]. While inorganic intercalation-type materials have been widely studied, they often suffer from inherent drawbacks such as structural collapse and sluggish ion diffusion, which limit their cycling stability and rate capability. In contrast, organic cathodes have emerged as highly compelling candidates for advanced AZIBs due to their lightweight nature, resource sustainability, structural flexibility, and environmental friendliness.

**Fig. 1:**
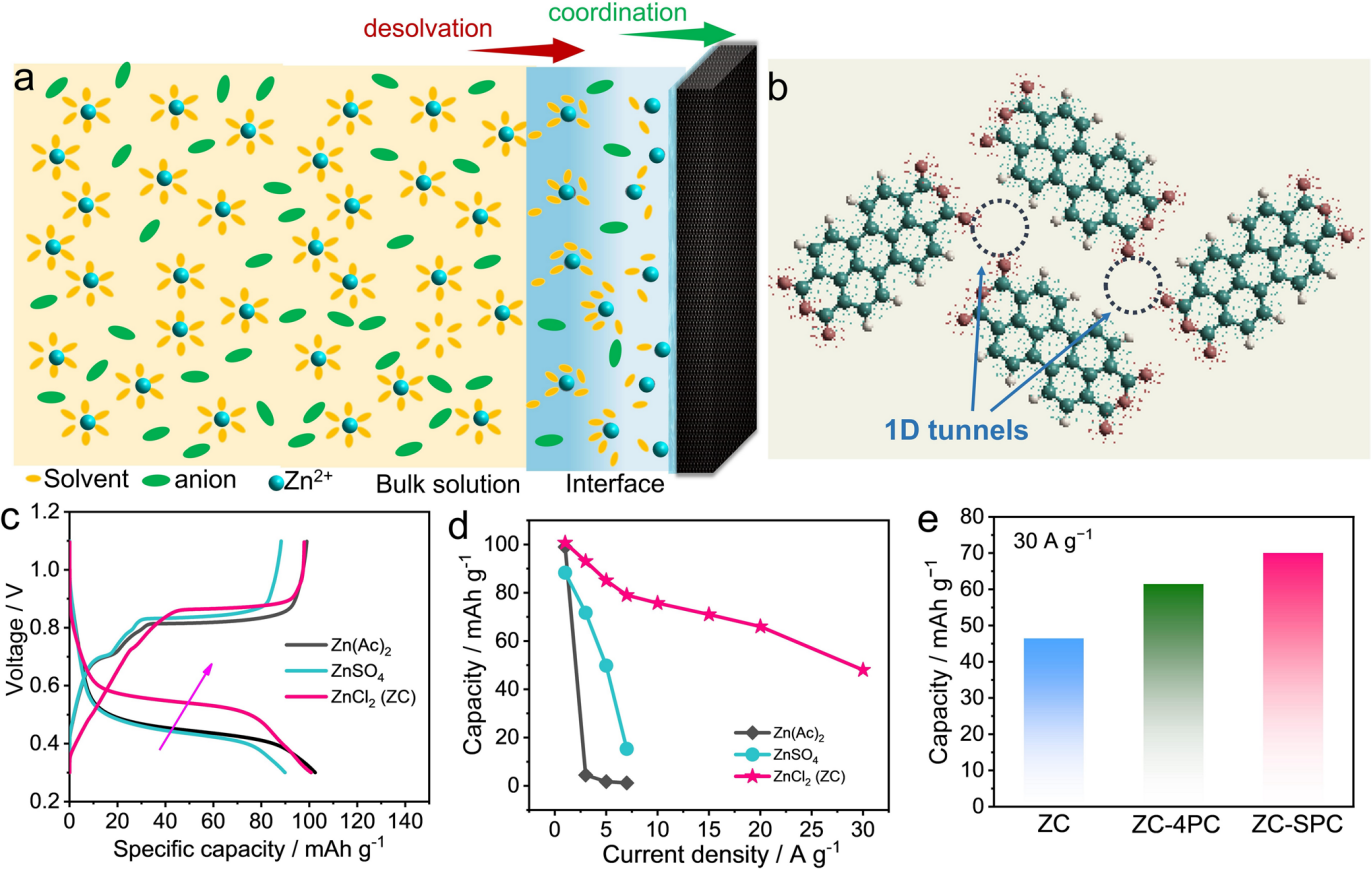
**a** Schematic illustration of the reaction process in organic cathodes. **b** Schematic diagram of the PTCDA structure. **c** GCD curves at 1 A g^−1^ and **d** rate capability of Zn//PTCDA batteries using different Zn salt aqueous solutions as electrolytes. **e** Specific capacity of Zn//PTCDA batteries using different ZnCl_2_-KCl aqueous solutions as electrolytes at 30 A g^−1^

The organic cathode charge storage process primarily involves the reversible redox coupling of Zn^2+^ with C=O, C=N, and C≡N groups, instead of conventional host–lattice intercalation (Fig. 1a), which can effectively mitigate material volume changes and fragmentation [22–24]. Consequently, organic materials with rich −C=O, −C=N, or C≡N groups are designed to achieve high-capacity and fast charge storage. Among them, 3,4,9,10-perylenetetracarboxylic dianhydride (PTCDA), as a typical aromatic organic molecular crystal, features a layered arrangement of planar molecules within its unit cell. These layers further stack along the third dimension, forming one-dimensional molecular tunnels (Fig. 1b) [25–27], which has demonstrated promising potential for application in AZIBs. As a notable example, by enhancing π-π stacking interactions, PTCDA could achieve a high capacity of 122.9 mAh g^−1^ at 0.2 A g^−1^ for Zn^2+^ storage with a 68.2% capacity retention after 1000 cycles [28]. However, a critical challenge for PTCDA and many small organic molecules is their tendency to dissolve in aqueous electrolytes, which triggers severe capacity fading. Although polymerization strategies can mitigate the dissolution problem, the complexity of the synthesis process limits large-scale application [29, 30]. Another alternative approach is electrolyte modification, such a “water-in-salt” electrolytes (WiSE), which have been successfully used to suppress dissolution in systems like polysulfides and vanadium oxides [31–34]. Yet, the high cost and compromised ionic conductivity of WiSE remains a significant concern. Therefore, developing low-cost and readily available electrolytes to inhibit the dissolution of organic electrodes, especially small organic molecules, is of paramount importance.

In this study, we demonstrate a rational electrolyte design by introducing KCl as a co-solute into a 2 M ZnCl_2_ aqueous electrolyte to achieve extended lifespan and superior rate capability for Zn//PTCDA battery. Firstly, experimental studies and molecular dynamics (MD) simulations illustrated that KCl co-solute effectively boosts the ionic conductivity of the electrolyte (from 53.6 to 109 mS cm^−1^) and modulates the solvation structure of Zn^2+^ (from Zn(H_2_O)_6_^2+^ to [Zn(H_2_O)_2_Cl_4_]^2−^), which significantly improves the ion diffusion kinetics by lowering the Zn^2+^ desolvation energy barrier (from 66.51 to 38.14 kJ mol^−1^). Secondly, the high ionic strength of KCl induces a “salting-out” effect, while the reshaped solvent network reduces water activity, collectively suppressing the dissolution of PTCDA molecules. Therefore, the fabricated Zn//PTCDA battery employing this modified electrolyte exhibits exceptional electrochemical performance, delivering a high capacity of 124.7 mAh g^−1^ (approximately 91.7% of the theoretical capacity) at 1 A g^−1^ (average voltage of 0.65 V), retaining 90.9% of its capacity after 10,000 cycles at 10 A g^−1^, and showcasing excellent rate capability (56% capacity retention at 30 A g^−1^). Notably, ex situ XPS, FT-IR analyses, and density functional theory (DFT) unveil a reversible Zn^2+^/K^+^ co-storage mechanism, where K^+^ acts as a charge shield and structural pillar, decreasing the ion migration energy barrier, buffering the substantial lattice strain induced by Zn^2+^ intercalation, and enabling rapid ion transport. This work not only provides fundamental insights into dual-ion storage mechanisms but also paves a practical pathway for developing durable and high-power Zn-organic batteries through ingenious electrolyte engineering.

## Experimental Section

### Formulation of the Electrolytes

The 2 M ZnCl_2_ electrolyte was obtained by dispersing ZnCl_2_ powder within deionized (DI) water. Similarly, the 2 M ZnSO_4_ solution was formulated by solubilizing ZnSO_4_ in DI water. Zn(Ac)_2_ (1.5 M) electrolyte was prepared by dissolving Zn(Ac)_2_ into DI water. The electrolytes, consisting of 2 M ZnCl_2_ with varying concentrations of KCl, were prepared by adding specific amounts of KCl solution (2 M, 4 M, or saturated) to a base 2 M ZnCl_2_ solution in deionized water.

### Materials Characterization

Field emission scanning electron microscopy (FE-SEM, JSM-6330F) was conducted to reveal the morphological and structural details of the materials. To elucidate the chemical bonds within the electrolytes, as well as to quantify the K content and Zn valence states, X-ray photoelectron spectroscopy (XPS, NEXSA, ThermoVG) was performed. Complementary structural insights were acquired through Raman spectroscopy (inVia, 532 nm laser) and Fourier transform infrared spectroscopy (FT-IR, Nicolet/Nexus 670). Prior to ex situ characterization, the cycled electrodes were retrieved at designated potentials and adequately washed with distilled water to strip away any residual electrolyte. The carbon (C) content of the electrolyte was determined using a Leco CHNS628O analyzer. The K^+^ and Zn^2+^ content in the discharged PTCDA cathode was determined using inductively coupled plasma mass spectrometry (ICP-MS, Agilent ICP-MS 7700).

### Electrochemical Measurements

To prepare the cathode slurry, PTCDA, acetylene black, and polyvinylidene fluoride (PVDF) binder (all analytical grade, Sigma-Aldrich) were dispersed in N-methyl-2-pyrrolidone (NMP, Sigma-Aldrich) at a mass ratio of 8:1:1. This homogeneous mixture was subsequently cast onto carbon paper and dried under vacuum at 60 °C for 12 h. The active material mass loading was controlled at 2–2.5 mg cm^–2^. Electrochemical evaluations, encompassing linear sweep voltammetry results (LSV), cyclic voltammetry (CV), galvanostatic charging/discharging (GCD), and electrochemical impedance spectra (EIS), were conducted using a CHI 760E workstation, with the EIS frequency swept from 10^–2^ to 10^5^ Hz.

### Calculation of the Diffusion Coefficient Based on EIS Data

The EIS method derives ${D}_{{\mathrm{Z}\mathrm{n}}^{2+}}$ based on the semi-infinite diffusion impedance model of a planar electrode, as well as Fick’s first and second laws. The specific derivation is as follows. According to the semi-infinite diffusion impedance model of a planar electrode, the Warburg impedance Z_ω_ can be expressed as:

1 $$z_{\omega } = \sigma \omega^{ - 1/2} - i\sigma \omega^{ - 1/2}$$

In Eq. (1), *σ* is the Warburg coefficient independent of concentration; *ω* is the angular frequency; *Z*′ is the real part of the impedance; ii is the imaginary unit; and *Z*′′ is the imaginary part of the impedance. Regarding Fick’s second law, by combining it with Fick’s first law, the impedance formula under EIS test conditions, the Butler–Volmer equation, and the calculation formula for the Warburg coefficient σ can be obtained:

2 $$D_{{Zn^{2 + } }} = \frac{1}{2} \left[ {\left( {\frac{{V_{m} }}{{Z_{i} {\mathrm{FS}}\sigma }}} \right)\left( {\frac{{{\mathrm{dE}}}}{{{\mathrm{dn}}}}} \right)} \right]^{2}$$

In Eq. (2), *V*_*m*_ is the molar volume of the active material; *E* is the open-circuit potential; *n* is the zinc ion content in the active material; and *Z*_*i*_ is the charge number of the diffusing ion.

### Computational Methods

Molecular dynamics (MD) simulations were conducted to investigate the structural and dynamic properties of Zn^2+^ ions in two aqueous electrolyte systems: (1) a 2 M ZnCl_2_ solution and (2) a mixed-salt solution containing 2 M ZnCl_2_ and 5.5 M KCl. All simulations were executed using the GROMACS package [35]. The systems were first energy-minimized via the steepest descent algorithm, followed by equilibration in the isothermal–isobaric (NPT) ensemble at 298 K and 1 bar for 5 ns, employing the v-rescale thermostat and the Parrinello–Rahman barostat [36, 37]. Production runs were then performed in the canonical (NVT) ensemble for 2 ns at 298 K with a 2.0-fs time step. Mean squared displacement (MSD) of Zn^2+^ ions vs. time was calculated, and diffusion coefficient (D) was derived from the long-time slope of the MSD using the Einstein relation. First-principles calculations based on density functional theory (DFT) were performed using the CP2K package, where the exchange–correlation functional was treated within the generalized gradient approximation (GGA) using the Perdew–Burke–Ernzerhof (PBE) parameterization [38]. A cluster model containing a representative molecular fragment and a [Zn(H_2_O)_2_Cl_4_]^2−^ cluster was constructed in a large simulation cell to avoid periodic interactions. The geometry was fully optimized using the BFGS algorithm. The migration pathway of the Zn hydrate between two stable sites was investigated using the Nudged Elastic Band (NEB) method [39]. The solvent effects were incorporated using an implicit solvation model [40]. The total energy of the fully optimized [Zn(H_2_O)_2_Cl_4_]^2−^ cluster in the implicit solvent was calculated. Subsequently, one water molecule from the first solvation shell was removed, and the resulting [Zn(H_2_O)_2_Cl_4_]^2−^ and [Zn(H_2_O)_6_]^2+^ system and the isolated water molecule were re-optimized separately in the same implicit solvent.

## Results and Discussion

We first studied the effects of different zinc salts on the discharge voltage and energy storage capacity of Zn//PTCDA batteries. Three low-cost and widely used Zn salts, namely, zinc chloride (2 M ZnCl_2_, denoted as ZC), zinc sulfate (2 M ZnSO_4_), and zinc acetate (1.5 M Zn(Ac)_2_), were chosen for comparison to assess the anion-dependent electrochemical behavior. The electrochemical performances of the Zn//PTCDA batteries in these electrolytes were characterized by galvanostatic charging/discharging (GCD) tests. As shown in Fig. 1c, the batteries assembled with different Zn salt electrolytes exhibit negligible differences in specific capacity, indicating that the capacity is primarily governed by the PTCDA cathode material. However, the discharge voltage of the ZC-based battery (0.55 V) is significantly higher than those using ZnSO_4_ and Zn(Ac)_2_ (both approximately 0.45 V), suggesting a notable influence of the anion species on the operating potential. The rate capacity of Zn//PTCDA batteries with different Zn salt electrolytes was further scrutinized, as presented in Fig. 1d. The ZC-based battery displays the best rate performance among the three, followed by ZnSO_4_ and Zn(Ac)_2_. Specifically, at a high current density of 10 A g^−1^, the capacity of the ZC-based battery is 79.0 mAh g^−1^, considerably higher than those of ZnSO_4_ and Zn(Ac)_2_ (15.3, 1.2 mAh g^−1^). Besides, the ZC-based battery maintains a capacity of 48.0 mAh g^−1^ even at a high current density of 30 A g^−1^, demonstrating its superior kinetics. Given that Cl^−^ anions are known to modulate ion solvation, we hypothesized that their presence might reorganize the electrolyte structure, thereby boosting kinetics. The boosted discharge voltage and rate performance of ZC-based battery can be primarily attributable to the presence of Cl^−^ anions. Cl^−^ ions participate in the solvation structure of Zn^2+^, facilitating the generation of complexes such as [Zn(H_2_O)_2_Cl_4_]^2−^, which reduces the desolvation energy barrier and improves ion diffusion kinetics. To further elucidate the role of Cl^−^ anions in modulating the voltage and rate performance, we introduced KCl solutions at varying concentrations (2 M, 4 M, and saturated) into the ZC electrolyte system. KCl was chosen as a co-solute due to its high solubility and ability to provide abundant Cl^−^ ions without introducing complex side reactions. This design aimed to systematically increase the Cl^−^ content while maintaining a constant Zn^2+^ concentration. As anticipated, the Zn//PTCDA battery with ZC electrolyte containing saturated KCl (denoted as ZC-SPC) exhibited a significantly higher discharge voltage compared to those using pure ZnCl_2_ or ZnCl_2_ with 4 M KCl (denoted as ZC-4PC), as supported by the GCD profiles in Fig. S1. The capacity trend among these electrolytes remained consistent with the initial salt comparison, even at 30 A g^−1^ (Fig. 1e), where the ZC-SPC system sustained the highest capacity. These results indicate that Cl^−^ anions in the electrolyte are pivotal for battery property.

**Fig. 2:**
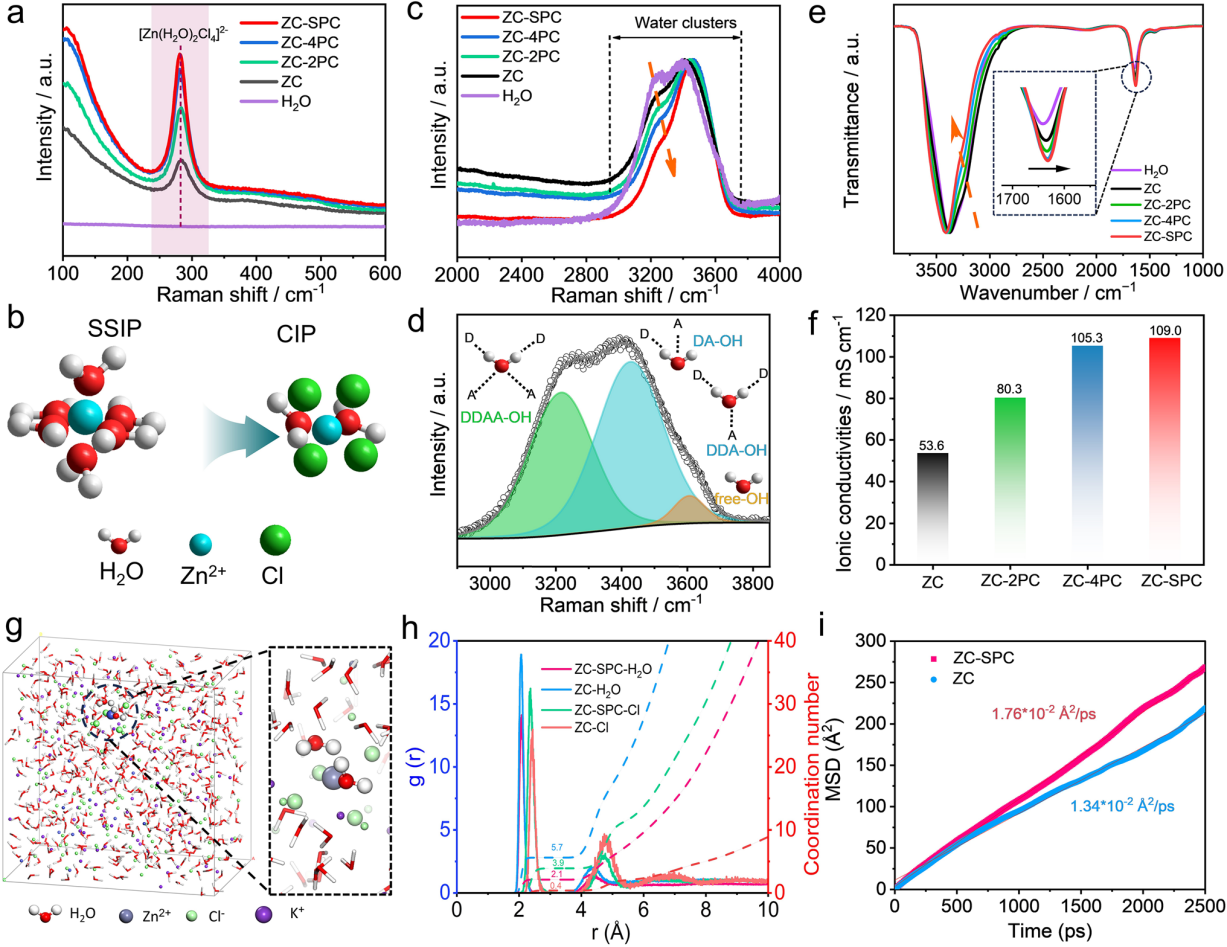
**a, c** Raman spectra of ZC, ZC-2PC, ZC-4PC, ZC-SPC, and H_2_O. **b** Molecular geometries of SSIP and CIP. **d** Raman O–H stretching band of water deconvolution. **e** FT-IR spectra of the electrolytes. **f** Ionic conductivities of the electrolytes. **g** 3D configuration of the ZC-SPC electrolyte obtained from MD simulations, with a magnified view highlighting the inner solvation structure of Zn^2+^. **h** RDF and the corresponding average coordination number of ZC and ZC-SPC. **i** Zn^2+^ ion self-diffusion coefficients from the simulated mean squared displacement (MSD) versus time curves for ZC and ZC-SPC electrolytes

To unravel the underlying mechanism behind this Cl^−^-mediated enhancement, we probed the evolution of the solvation structure in ZC electrolytes with different KCl concentrations using Raman spectroscopy, a powerful technique for identifying molecular vibrations and coordination environments. Figure 2a shows the typical Raman spectra of these electrolytes. The Raman band observed around 282 cm^−1^ in all ZnCl_2_-based electrolytes is characteristic of the symmetric stretching vibration of Zn–Cl bonds in a tetrachlorozincate-like coordination environment, as typified by complexes such as [Zn(H_2_O)_2_Cl_4_]^2−^. This provides direct spectroscopic evidence for the presence of Zn^2+^ species with significant chloride coordination in these electrolytes [41]. Notably, the intensity of this peak displays a gradual enhancement with increasing KCl concentration (Fig. 2b), indicating that elevated Cl^−^ content promotes the formation of these Cl^−^-rich complexes. This structural alteration directly impacts the aqueous environment, as the formation of [Zn(H_2_O)_2_Cl_4_]^2−^reduces the primary hydration number of Zn^2+^, releasing them into the bulk electrolyte and disrupting the original hydrogen-bonded water network [42]. The profound impact of this solvation shell restructuring on the aqueous environment is clearly revealed in the O–H stretching vibration region (Fig. 2c, d). As the electrolyte composition shifts from ZC to ZC-SPC, the relative proportion of the coupled O–H stretching vibration peak (around 3220 cm^−1^) gradually decreases. This peak is characteristic of water molecules engaged in a fully hydrogen-bonded, tetrahedral-like configuration (DDAA-OH, double donor–double acceptor), and its diminution indicates the disintegration of bulk-like water clusters. Concurrently, the contribution of a component at higher wavenumbers (above 3400 cm^−1^), attributed to water molecules with weaker or incomplete hydrogen bonding (DA-OH, single donor–single acceptor; DDA-OH, double donor–single acceptor), shows a marked increase. The proportion of another stretching peak at higher wavenumbers (above 3400 cm^−1^), corresponding to water molecules with weaker or incompletely hydrogen bonding (DA and DDA types), shows a noticeable increase. Such change is ascribed to the formation of Cl^−^···H–O interactions in ZC-SPC, which partially replace conventional O–H···O bonds [43]. These modified intermolecular forces reorganize the solvent structure, facilitating the formation of fragmented water–salt oligomers by connecting halogen anions and water molecules.

Fourier transform infrared (FT-IR) spectroscopy was employed to further corroborate the structural evolution of the electrolyte solvation environment. As presented in Fig. 2e, the wide peak located at 3200–3400 cm^−1^ is assigned to the O–H stretching vibration of solvated H_2_O and exhibits distinct changes with increasing KCl concentration. The intensity of the symmetric stretching vibration (around 3200 cm^−1^) weakens, while the asymmetric stretching vibration (near 3400 cm^−1^) intensifies, indicating that strong ion–dipole interactions between Zn^2+^/Cl^−^ and water molecules disrupt the original O–H hydrogen-bonding network. Moreover, the H–O–H bending vibration peak shifts from 1635 to 1631 cm^−1^ as the KCl concentration increases from 2 M to saturated, suggesting enhanced ion–solvent interactions and an increase in solution viscosity. Interestingly, despite the expected increase in viscosity that typically impedes ion mobility, the ionic conductivity presents a substantial enhancement from 53.6 to 109 mS cm^−1^ upon KCl addition (Fig. 2f). Even at − 20 °C, the ZC-SPC electrolyte retains a notable ionic conductivity of ~ 13.3 mS cm^−1^, demonstrating far superior conductivity retention at low temperatures compared to the baseline system. This apparent paradox highlights the crucial role of KCl in fundamentally altering ion transport mechanisms. The significantly improved conductivity may be attributed to the reduced de-solvation energy barrier and the formation of a more fluid-like, fragmented solvent structure that facilitates faster ion migration. Molecular dynamics (MD) simulations were performed to gain further insight into the Zn^2+^ solvation structure. The 3D configuration of the ZC and ZC-SPC electrolyte obtained from MD simulation is shown in Fig. S2. Figures 2g and S2 show that averagely each Zn^2+^ is ligated by six H_2_O molecules. However, as observed in Fig. 2g, four coordinated H_2_O molecules are substituted by four Cl^−^ in the ZC-SPC electrolyte. Additionally, the radial distribution functions (RDFs) and coordination number (CN) distributions were calculated to provide a quantitative analysis of the Zn^2+^ solvation shell. For the ZC electrolyte, a peak attributed to the Zn–O(H_2_O) pair is observed at approximately 0.20 nm, corresponding to the primary solvation shell with an average coordination number of 5.7. The peak for the Zn–Cl pair appears at around 0.24 nm with an average coordination number of 0.4 (Figs. 2h and S2). In contrast, for the ZC-SPC electrolyte, while the Zn–O peak persists near 0.20 nm, the average number of coordinated H_2_O molecules decreases significantly to 2.1. Concurrently, a sharp peak corresponding to the Zn–Cl pair emerges at 0.23 nm, with the average Cl^−^ coordination number increasing to 3.9. This confirms the successful insertion of Cl^−^ into the Zn^2+^ solvation sheath, leading to the formation of (Zn(H_2_O)_2_Cl_4_)^2−^ (Fig. 2h). Moreover, the self-diffusion coefficients of the two electrolyte systems were compared through theoretical calculations. It was found that the self-diffusion coefficient of the ZC-SPC electrolyte system (1.76 × 10^−2^ Å^2^ ps^−1^) is superior to that of the ZC electrolyte system (1.34 × 10^−2^ Å^2^ ps^−1^) (Fig. 2i), indicating that the introduction of KCl increases the self-diffusion coefficient and simultaneously enhances the transport efficiency of Zn^2+^. Furthermore, this value is significantly greater than those of ZnSO_4_ (4.4 × 10^−3^ Å^2^ ps^−1^) [44] and Zn(Ac)_2_ (1.61 × 10^−4^ Å^2^ ps^−1^) [45].

Beyond the bulk transport properties, the interfacial characteristics of the electrolytes were also investigated. The pH value increases from 4.88 to 5.53 with KCl addition (Fig. S3), creating a less acidic environment that can effectively suppress hydrogen evolution side reactions at the anode. To verify the inhibitory effect of KCl on the hydrogen evolution side reaction, we compared the electrochemical behavior of the ZC-SPC electrolyte with that of the pure ZC electrolyte. Linear sweep voltammetry results (LSV) show that (Fig. S4a), compared with the pure ZC electrolyte, the hydrogen evolution current on the zinc anode is significantly reduced. Meanwhile, linear polarization Tafel testing (Fig. S4b) indicates that the corrosion potential (Ecorr) shifts from − 1.03 to − 0.97 V, demonstrating that the high concentration of KCl effectively retards the self-corrosion process of zinc in this system. Together, these results confirm that the introduction of a high concentration of KCl markedly suppresses the hydrogen evolution side reaction. Simultaneously, the introduction of KCl markedly improves the electrolyte wettability on the PTCDA electrode, as evidenced by the contact angle decreasing from 110° in the ZC electrolyte to 65° in the ZC-SPC electrolyte (Fig. S5). This enhanced wettability reduces interfacial energy and promotes a more uniform Zn^2+^ flux across cathode surface during charge/discharge cycles, thereby promoting reaction kinetics and stability [46]. Based on these comprehensive results, the ZC-SPC electrolyte was selected as the optimal system for further electrochemical evaluation.

**Fig. 3:**
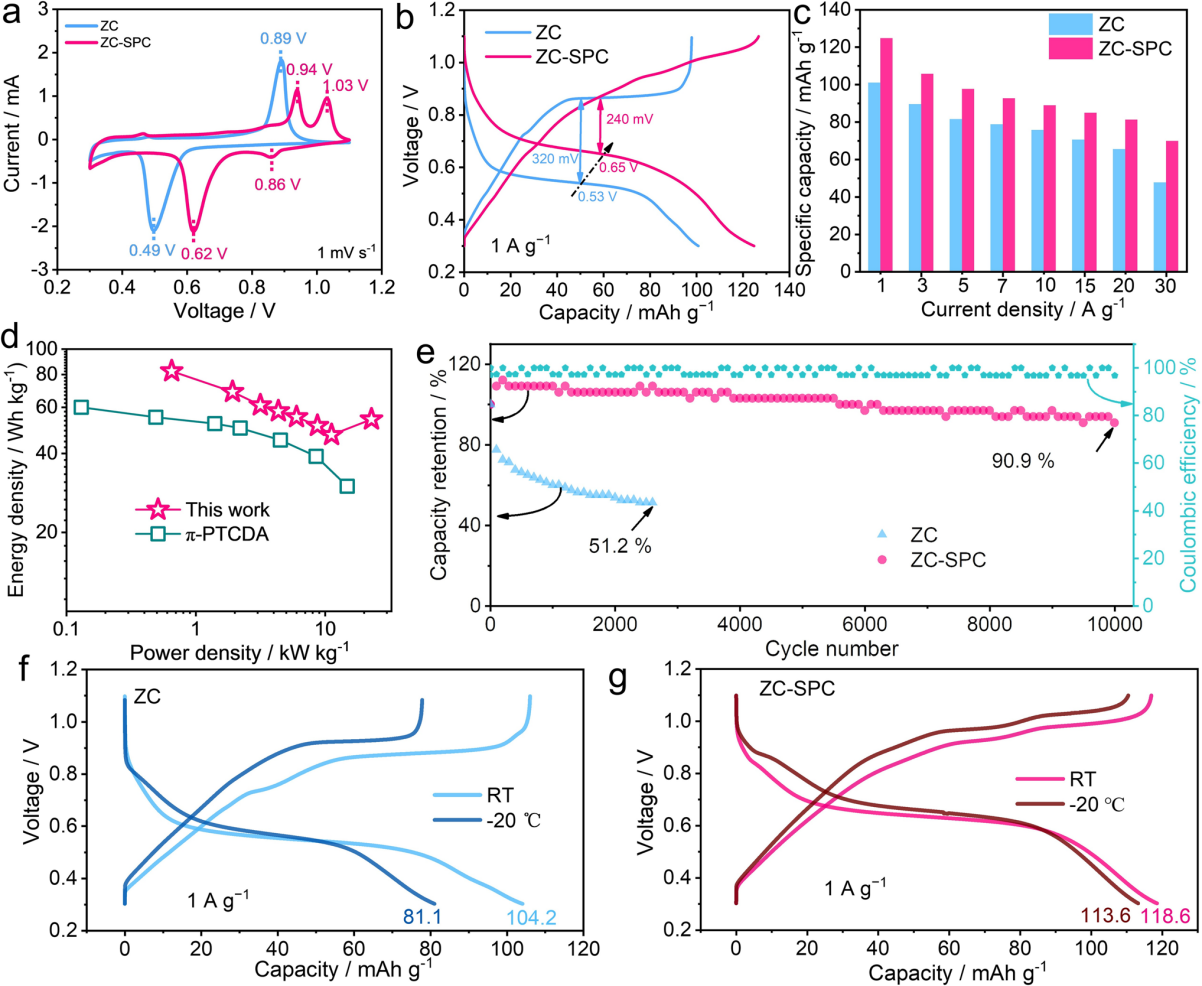
**a** CV curves at 1 mV s^−1^, **b** GCD profiles at 1 A g^−1^, **c** rate performance of Zn//PTCDA batteries based on ZC and ZC-SPC electrolytes. **d** Comparison of energy density and power density of Zn//PTCDA with ZC-SPC electrolyte and Zn//π-PTCDA battery. **e** Cycling stability comparison of Zn//PTCDA batteries based on ZC and ZC-SPC electrolytes. The GCD curves of Zn//PTCDA batteries with **f** ZC and **g** ZC-SPC electrolytes at room temperature and − 20 °C

To test the practical application of the KCl-modified electrolyte, we examined the electrochemical properties of Zn//PTCDA batteries. Initial insights into the redox behavior were obtained from CV measurements. The battery with ZC-SPC electrolyte exhibits two distinct redox peaks, whereas only a single redox peak is observed in the ZC system. This suggests that the addition of KCl obviously alters the redox behavior of the PTCDA cathode, which will be discussed in detail later (Fig. 3a). Figures 3b and S6 display the galvanostatic charge–discharge (GCD) profiles for the two batteries at 1 and 30 A g^−1^, respectively. At the current density of 1 A g^−1^, the capacity of Zn//PTCDA battery employing ZC-SPC electrolyte is 124.7 mAh g^−1^ (~ 91.7% theoretical capacity), higher than that of ZC electrolyte (97.9 mAh g^−1^). The performance markedly exceeds the values reported for previously developed Zn//π-PTCDA batteries [28] and other batteries (Table S1). The Zn//PTCDA battery employing ZC-SPC electrolyte shows remarkably reduced polarization, with the voltage hysteresis decreasing from 320 to 240 mV at 1 A g^−1^ and from 627 to 235 mV at 30 A g^−1^, indicating significantly lowered energy barriers for ion insertion/extraction during the energy storage process. Furthermore, average discharge voltage increases from 0.53 V for ZC to 0.65 V for ZC-SPC, contributing to improved energy density. The rate capability was rigorously assessed by incrementing the current density (1 − 30 A g^−1^), with corresponding results depicted in Figs. 3c and S7. Furthermore, we found that the discharge plateau approaches approximately 0.8 V at a high current density of 30 A g^−1^. To elucidate the formation mechanism of this plateau, we conducted CV measurements at higher scan rates of 60 and 80 mV s^−1^ (Fig. S8). The results show that in the ZC-SPC electrolyte, the peak originally located at around 0.6 V gradually diminishes or even disappears, while the peak signal near 0.8 V progressively strengthens and eventually becomes dominant. To further investigate the nature of the electrochemical reaction at this potential, we performed control LSV tests in pure saturated KCl electrolyte and ZC-SPC electrolyte (Fig. S9), which revealed a clear peak at nearly the same potential (corresponding to approximately 0.8 V in the battery configuration). In addition, inductively coupled plasma (ICP) and scanning electron microscopy—energy-dispersive X-ray spectroscopy mapping (SEM-mapping) analyses indicate that under a high current density of 30 A g^−1^, the K^+^ content in the discharged PTCDA cathode is significantly higher than that of Zn^2+^ (Fig. S10). Collectively, these results provide evidence that the electrochemical response at this potential originates from K^+^ ion intercalation/deintercalation processes. The underlying reason may be attributed to the fact that Zn^2+^, due to its + 2 charge, exhibits stronger electrostatic interactions with coordinated water molecules, resulting in a tighter hydration shell and a higher coordination number (CN=6). Consequently, its Stokes hydration radius (4.3 Å) is larger than that of K⁺ (3.3 Å), leading to a lower bulk diffusion coefficient for Zn^2+^ compared to K^+^. Therefore, under high current density, the faster transport capability of K^+^ enables it to serve as the primary charge carrier, thereby dominating the discharge process [47, 48]. Specifically, the Zn//PTCDA battery with ZC-SPC maintains superior capacity retention across all rates, delivering 70 mAh g^−1^ (56% retention) under 30 A g^−1^, which contrasts sharply with rapid capacity degradation observed in the ZC system. This exceptional rate performance translates to an outstanding power density (22.8 kW kg^−1^), as calculated from GCD data, which substantially surpasses the values reported for previously developed Zn//π-PTCDA batteries [28] (Fig. 3d).

Long-term cycling stability represents another critical performance metric. As illustrated in Figs. 3e, S11, and S12, battery cycling stability improves with rising KCl concentration. Upon the KCl concentration reaches saturation, the battery exhibits the best long-term cycling stability. The ZC-SPC battery demonstrates outstanding cycling stability, maintaining 90.9% and 93.6% of its initial capacity after 10,000 cycles at 10 and 30 A g^−1^, respectively. In stark contrast, the ZC electrolyte suffers rapid capacity decay, retaining only 51.2% after a mere 2500 cycles. By overlaying the GCD curves from different cycles (Fig. S13), it can be clearly observed that for the battery with the ZC-SPC electrolyte, the charge and discharge voltage plateaus remain highly coincident even after thousands of cycles, with only minimal increase in overpotential. The electrochemical impedance spectra (EIS) and X-ray diffraction (XRD) of the batteries before and after cycling were also examined (Figs. S14 and S15). For the Zn//PTCDA battery with the ZC-SPC electrolyte after cycling, the charge transfer resistance increases only slightly, and the diffusion resistance indicated by the Warburg region remains at a relatively low level (Fig. S14). The XRD patterns reveal that the main peaks of cycled PTCDA are in agreement with those of pristine material, with no noticeable new peaks that can be assigned to secondary phases (Fig. S15). Furthermore, at 0.1 A g^−1^ (Fig. S16), the slower charge–discharge process significantly extends contact time between electrode material and electrolyte. This amplifies impact of any potential side reactions, including but not limited to gradual PTCDA dissolution, electrolyte decomposition, and accumulation of interfacial by-products, thereby making the test conditions more stringent. Under these conditions, the capacity retention of the batteries with the ZC-SPC electrolyte remains notably better than that of ZC-based battery. The enhanced stability is further corroborated by a 7-day electrode soaking test (Fig. S17), no noticeable color change is observed in the ZC-SPC electrolyte, whereas distinct yellow coloration appears in the ZC electrolyte. Furthermore, we employed an elemental analyzer to accurately measure the carbon content in the filtered electrolytes. The results show that the carbon content in the saturated ZC-SPC electrolyte is significantly lower, specifically about half that in the ZC electrolyte. These indicates effective suppression of PTCDA dissolution, which is attributable to the “salting-out” effect induced by the high ionic strength of KCl and the reduced water activity caused by the restructured solvent network. Additionally, we also investigated the low-temperature adaptability of the Zn//PTCDA batteries with different electrolytes. Interestingly, the KCl co-solute was found to lower the freezing point of the ZC electrolyte, allowing it to remain in a liquid state even at − 20 °C (Fig. S18). Figure 3f, g compares the low-temperature performance of Zn//PTCDA batteries using ZC and ZC-SPC electrolytes, respectively. The battery with ZC-SPC electrolyte exhibited 96% capacity retention at − 20 °C, significantly outperforming the 78% retention of the ZC electrolyte. We also tested the rate performance of Zn//PTCDA batteries with the ZC-SPC electrolyte at − 20 °C (Fig. S19). The batteries using ZC-SPC delivered a relatively high capacity of about 40 mAh g^−1^ at high current density of 30 A g^−1^. In contrast, the battery with ZC electrolyte fails to work at 30 A g^−1^. In contrast, the EIS measurements were carried out at room temperature and low temperature (− 20 °C) (Fig. S20). At room temperature, the electrochemical impedance of the ZC-SPC electrolyte is approximately 24 Ω. Under the harsh condition of − 20 °C, the ZC-SPC electrolyte exhibits an electrochemical impedance of 53 Ω. These excellent low-temperature performances underscore the advantage of the modified electrolyte in practical applications requiring environmental adaptability.

**Fig. 4:**
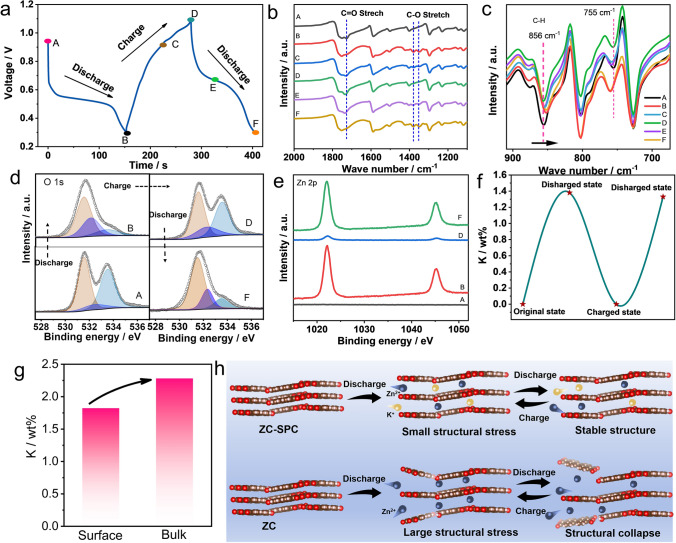
**a** GCD profiles at 1 A g^−1^. **b**, **c** Ex situ FT-IR, **d** XPS O 1*s*, and **e** XPS Zn 2*p* spectra at different charge/discharge states. **f** K content under different states of charge and discharge. **g** The content of K before and after sputtering. **h** Schematic diagram depicting the energy storage mechanism of PTCDA electrode within ZC and ZC-SPC electrolytes

For a clearer insight into the energy storage mechanism of PTCDA electrodes in ZC-SPC electrolyte, ex situ FT-IR and X-ray photoelectron spectroscopy (XPS) tests were conducted at strategically selected states during charge–discharge cycles, as outlined in Fig. 4a. Figure 4b and 4c shows the ex situ FT-IR spectra of PTCDA electrodes at various charging/discharging states. The intensity changes of all characteristic peaks are highly reversible throughout the cycling process. More specifically, during the discharging process (A → B, D → F), the vibration peak of C=O near 1723 cm^−1^ gradually disappears, whereas a new peak emerges near 1352 cm^−1^, corresponding to C–O vibration. This spectral evolution signifies the electrochemical transformation of C=O to C–O–Zn, associated with Zn^2+^ intercalation. Upon charging (B → D), C–O and C=O peak intensities recover to their initial state, confirming the reversible electrochemical reaction characteristics of PTCDA during the ion intercalation process. Additionally, reversible changes in the intensity and position of the C–H vibration peak were observed (Fig. 4c), further supporting the structural reversibility of PTCDA during cycling. The reversible conversion process was further verified by XPS analysis. As depicted in Fig. 4d, C=O peak intensity (at 533.5 eV) decreases during discharge process but increases again during charging, while C–O peak (at 532.1 eV) shows opposite trend. Quantitative analysis reveals that the Zn content in the discharged PTCDA cathode is approximately 12.4 times that in charged state, agreeing with the enhanced peak intensity attributed to the C–O–Zn complex and the ex situ FT-IR data. Notably, the reversible intercalation/deintercalation of *K*^+^ was also clearly observed. As illustrated in Fig. 4f, the *K* content is nearly zero in the charged state, but reaches 1.35% in the discharged state. To exclude the possibility of surface-adsorbed *K*, XPS was performed on a PTCDA electrode immersed solely in the ZC-SPC electrolyte without undergoing electrochemical cycling. The surface *K* signal of this soaked electrode was minimal (< 0.34%), significantly lower than that of the discharged state (Fig. S21). Additionally, XPS depth profiling was conducted on the discharged electrode. *K* signal in the discharged state originates from ion co-intercalation rather than physical adsorption or surface residues. As shown in Fig. 4g, the *K* content exhibited a slight increase rather than a decrease after sputtering, mirroring the trend observed for Zn (Fig. S22). This confirms the intercalation of K⁺ ions into the bulk structure of PTCDA. Furthermore, SEM–EDS analysis of the discharged cathode provided additional evidence for K^+^ co-intercalation. As shown in Fig. S23a, b, K is uniformly distributed across the active material. EDS spectroscopy analysis (Fig. S23c) revealed a *K* content of 1.49 wt%, which is consistent with the XPS data and further confirms the intercalation of K^+^ into the PTCDA structure. The synergistic Zn^2+^/K^+^ co-intercalation mechanism offers multiple advantages for battery performance. Firstly, the monovalent K^+^ ions, with their smaller hydrated radius, act as charge shields that partially neutralize negative charges on carbonyl groups, thereby reducing electrostatic repulsion for subsequent Zn^2+^ insertion. This charge shielding effect minimizes structural stress and volume changes, effectively suppressing cathode fragmentation and dissolution. Secondly, K^+^ ions serve as structural pillars that stabilize the layered architecture of PTCDA through non-covalent interactions, buffering the lattice strain induced by Zn^2+^ intercalation and preventing cyclic fatigue. Based on these comprehensive analyses, Fig. 4h illustrates the proposed energy storage mechanism of the PTCDA electrode within ZC and ZC-SPC electrolytes.

**Fig. 5:**
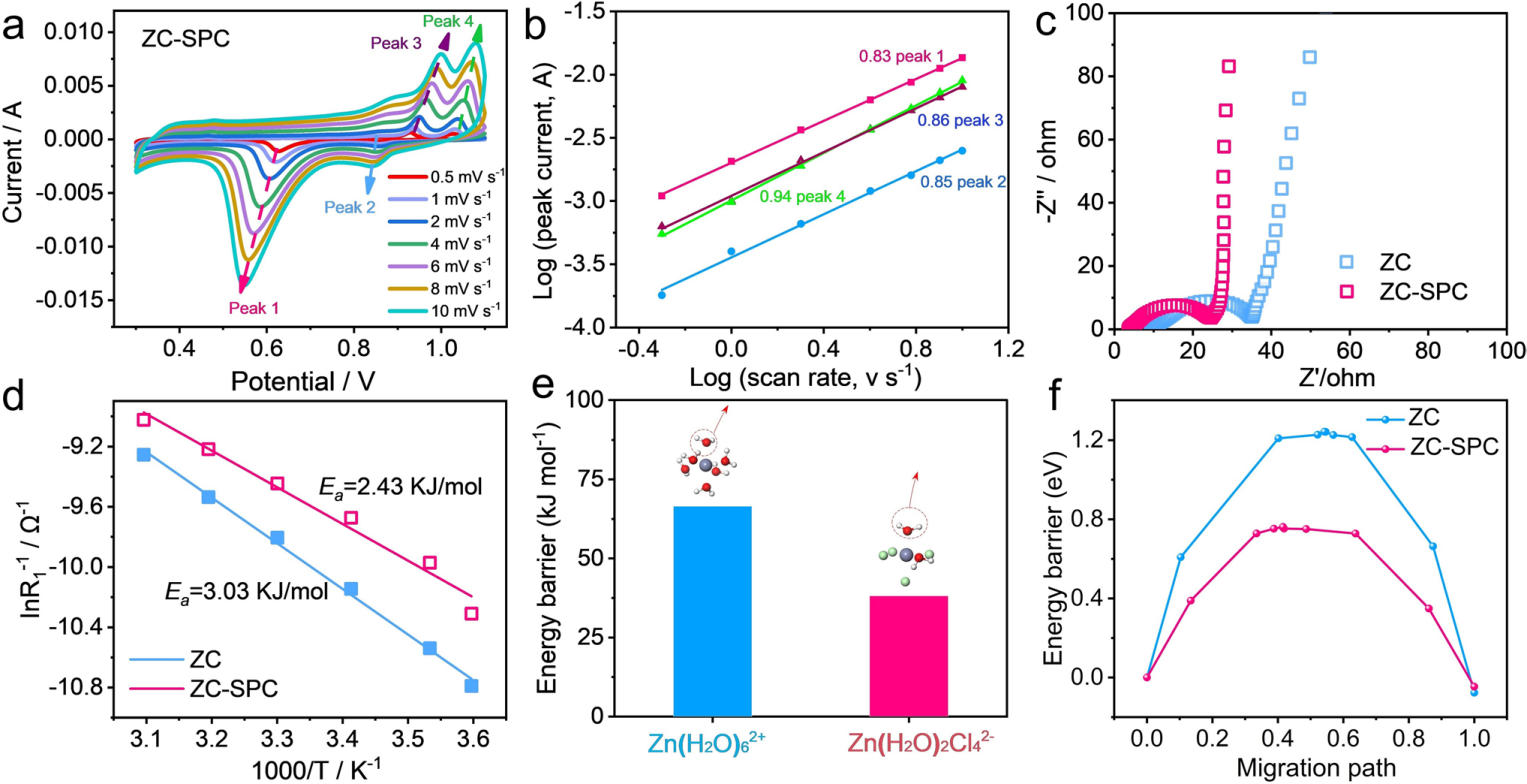
CV curves of Zn//PTCDA batteries under various scan rates within **a** ZC-SPC. **b** Log(*i*) versus log(*v*) plots of cathodic and anodic currents response at marked peaks in a. **c** EIS of Zn//PTCDA batteries in ZC and ZC-SPC. **d** Arrhenius behavior of the temperature-dependent charge transfer resistance of PTCDA in ZC and ZC-SPC electrolytes. **e** The desolvation energy of Zn^2+^ in ZC and ZC-SPC electrolytes. **f** The migration energy barrier of Zn^2+^ in PTCDA with ZC and ZC-SPC electrolytes

Having elucidated the Zn^2+^/K^+^ co-intercalation mechanism, we next explored the kinetic origins of the enhanced rate performance through electrochemical analysis. CV curves at various scan rates were measured to qualitatively analyze the capacitive contributions. In Fig. 5a, four redox peaks of the CV curves are labeled as Peaks 1–4, corresponding to different stages of the co-intercalation process. Peak 1 (~ 0.5 V, reduction peak) and Peak 3 (~ 0.9 V, oxidation peak) form a redox couple at relatively lower potentials. This couple primarily corresponds to the reversible redox reaction of Zn^2+^ in the PTCDA cathode material. Peak 2 (at approximately 0.8 V, reduction peak) and Peak 4 (at approximately 1.15 V, oxidation peak) form a redox couple at relatively higher potentials. This couple is likely associated with the reversible extraction/insertion of K^+^ in the PTCDA cathode material. The high concentration of K^+^ makes the intercalation of K^+^ into PTCDA possible. This process is associated with high K^+^ concentration in electrolyte and specific interactions between K^+^ and PTCDA framework. The slope (*b*) of the log(*i*) versus log(*v*) curves reflects the charge storage mechanism, where *b* approaching 1 indicates capacitive-dominated behavior and *b* approaching 0.5 suggests diffusion-dominated behavior [49]. Based on the fitting results from CV curves at different scan rates (Fig. 5b), the calculated *b*-values for peaks 1–4 in ZC-SPC electrolyte are 0.83, 0.85, 0.86, and 0.94, respectively, implying that K^+^-Zn^2+^ co-intercalation process involves both capacitive and diffusion-controlled mechanisms. All four b-values in the ZC-SPC electrolyte are higher than those in the pristine ZC electrolyte (Fig. S24). Furthermore, the electrode reactions in the ZC-SPC electrolyte system exhibit a significantly higher percentage of pseudocapacitive contribution. At 0.5 mV s^−1^ scan rate, battery pseudocapacitive contribution with the ZC-SPC electrolyte reaches 92.8%, notably exceeding that of the ZC electrolyte (89.2%) (Fig. S25). As the scan rate increases from 0.5 to 10 mV s^−1^, this pseudocapacitive contribution rises from 92.8% to 98.3% (Fig. S25), suggesting that the charge–discharge processes in the ZC-SPC electrolyte are more dominated by pseudocapacitive effects, thereby ensuring superior rate performance. Electrochemical impedance spectroscopy (EIS) provides additional insights into the kinetic advantages. Figure 5e presents the Nyquist plots of Zn//PTCDA batteries employing ZC and ZC-SPC electrolytes. The results reveal that both the ohmic resistance and charge transfer resistance (*R*_ct_) in the ZC-SPC electrolyte are lower than those in the ZC electrolyte. Furthermore, the calculated Zn^2+^ diffusion coefficient (${D}_{{\mathrm{Z}\mathrm{n}}^{2+}}$) is 8.44 × 10^−10^ cm^2^ s^−1^, significantly higher than that in pure ZC electrolyte (8.44 × 10^−12^ cm^2^ s^−1^) (Fig. S26). These results further confirm the critical role of KCl in promoting ion transport and electrochemical kinetics. To quantify the desolvation kinetics, we calculated the activation energy (*E*ₐ) for Zn^2+^ ion desolvation kinetics in both electrolytes based on the Arrhenius equation and EIS results at different temperatures (Figs. 5f and S27). The calculated *E*ₐ values for ZC and ZC-SPC electrolytes are 3.03 and 2.43 kJ mol^−1^, respectively, indicating a significantly lower desolvation energy barrier in the ZC-SPC system. To further validate the reduction in desolvation energy at the molecular level, density functional theory (DFT) calculations were performed to investigate the binding energies within the solvation sheaths. As shown in Figs. 5e and S28, the calculated binding energy between Zn^2+^ and the solvation shell in the ZC-SPC system is 38.14 kJ mol^−1^, substantially lower than 66.51 kJ mol^−1^ for ZC system. This reduction can be explained by the addition of saturated KCl, where Cl^−^ ions participate in competitive coordination to form numerous Zn–Cl bonds. The resulting chlorine-containing complex ion is a bulky anion with low charge density; the positive charge of the central metal is shielded and neutralized by the ligands, leading to very weak interactions with water molecules. This mechanism results in a looser solvation sheath, which ultimately facilitates Zn^2+^ storage and enhances overall kinetics. Consequently, the energy barrier for shedding solvent molecules during intercalation is minimized, corroborating the reduced activation energy observed in electrochemical measurements. Furthermore, the migration energy barriers within the PTCDA framework were investigated for both ZC and ZC-SPC electrolytes (Figs. 5f and S29). The energy barrier in the ZC electrolyte was found to be significantly higher than that in the ZC-SPC electrolyte (1.24 vs. 0.76 eV), demonstrating the remarkable ion diffusion kinetics facilitated by the ZC-SPC electrolyte, mainly explained by the co-intercalation of K^+^ ions into PTCDA bulk phase. The larger ionic radius of K^+^ alleviates steric hindrance within the PTCDA structure, thereby reducing the diffusion resistance for Zn^2+^. Collectively, these comprehensive kinetic analyses consistently demonstrate that the ZC-SPC electrolyte promotes a more capacitive-driven and less energy-barred reaction pathway, which is fundamental to the achieved high-rate performance and long-term cyclability.

## Conclusions

In summary, we have elucidated the effects of introducing KCl as a co-solute into ZnCl_2_ aqueous electrolytes on the electrochemical performance of PTCDA. Our study revealed that increasing the KCl concentration simultaneously enhances the specific capacity, operating voltage, and rate capability. Zn//PTCDA battery with the optimized ZC-SPC electrolyte delivers a high capacity 124.7 mAh g^−1^ with 0.65 V (average operation voltage). Furthermore, this battery also achieves exceptional cycling stability (90.9% capacity retention after 10,000 cycles) and remarkable rate capability (> 56% capacity retention at 30 A g^−1^). This superior performance originates from a fundamental alteration for Zn^2+^ solvation structure and a significant enhancement for conductivity. Forming a [Zn(H_2_O)_2_Cl_4_]^2−^-rich solvation structure in the ZC-SPC electrolyte nearly doubles the ionic conductivity and reduces the Zn^2+^ desolvation energy barrier, thereby significantly improving the ion diffusion kinetics. Additionally, the intercalated K^+^ can act as a charge shield and structural pillar, mitigating Zn^2+^ intercalation strain and facilitating rapid ion transport, thereby promoting the structural stability and rate performance of PTCDA. These findings provide an electrolyte design strategy based on multi-salt concentration regulation, which will advance the practical application of aqueous electrolytes and accelerate the advancement of cost-effective, long-life zinc metal batteries.

## Supplementary Information

Below is the link to the electronic supplementary material.

Supplementary file1 (DOCX 39.0 MB)
