# Supplementary material for: Dual-Ion Co-Storage via Solvation Structure Tuning Toward Ultrafast and Durable Zinc-Organic Batteries
Source: Nanomicro Lett. 2026 Jul 28;19:7. doi: 10.1007/s40820-026-02304-7 (PMC13415416; doi:10.1007/s40820-026-02304-7)
Supplement: Supplementary file 1 — Supplementary file1 (DOCX 39.0 MB) [file 40820_2026_2304_MOESM1_ESM.docx]

Supporting Information for

**Dual-Ion Co-Storage via Solvation Structure Tuning Toward Ultrafast and Durable Zinc-Organic Batteries**

Si Liu^1^, Zhifeng Lin^1^, Yanxia Yu^3,^ *, Haozhe Zhang^4,^ * and Xihong Lu^2,^ *

^1^ School of Electronic and Information Engineering, School of Environmental and Chemical Engineering, Foshan University, Foshan 528000, P. R. China

^2^ The Key Lab of Low-carbon Chem & Energy Conservation of Guangdong Province, School of Chemistry, Sun Yat-Sen University, Guangzhou 510275, P. R. China

^3^ School of Applied Physics and Materials, Wuyi University, Jiangmen 529020, P. R. China

^4^ Pritzker School of Molecular Engineering, University of Chicago, Chicago, IL 60637, USA

* Corresponding authors. E-mail: yuyx26@mail.sysu.edu.cn (Yanxia Yu); zhhaozhe@uchicago.edu (Haozhe Zhang); luxh6@mail.sysu.edu.cn (Xihong Lu)

**S1 Supporting Figures**


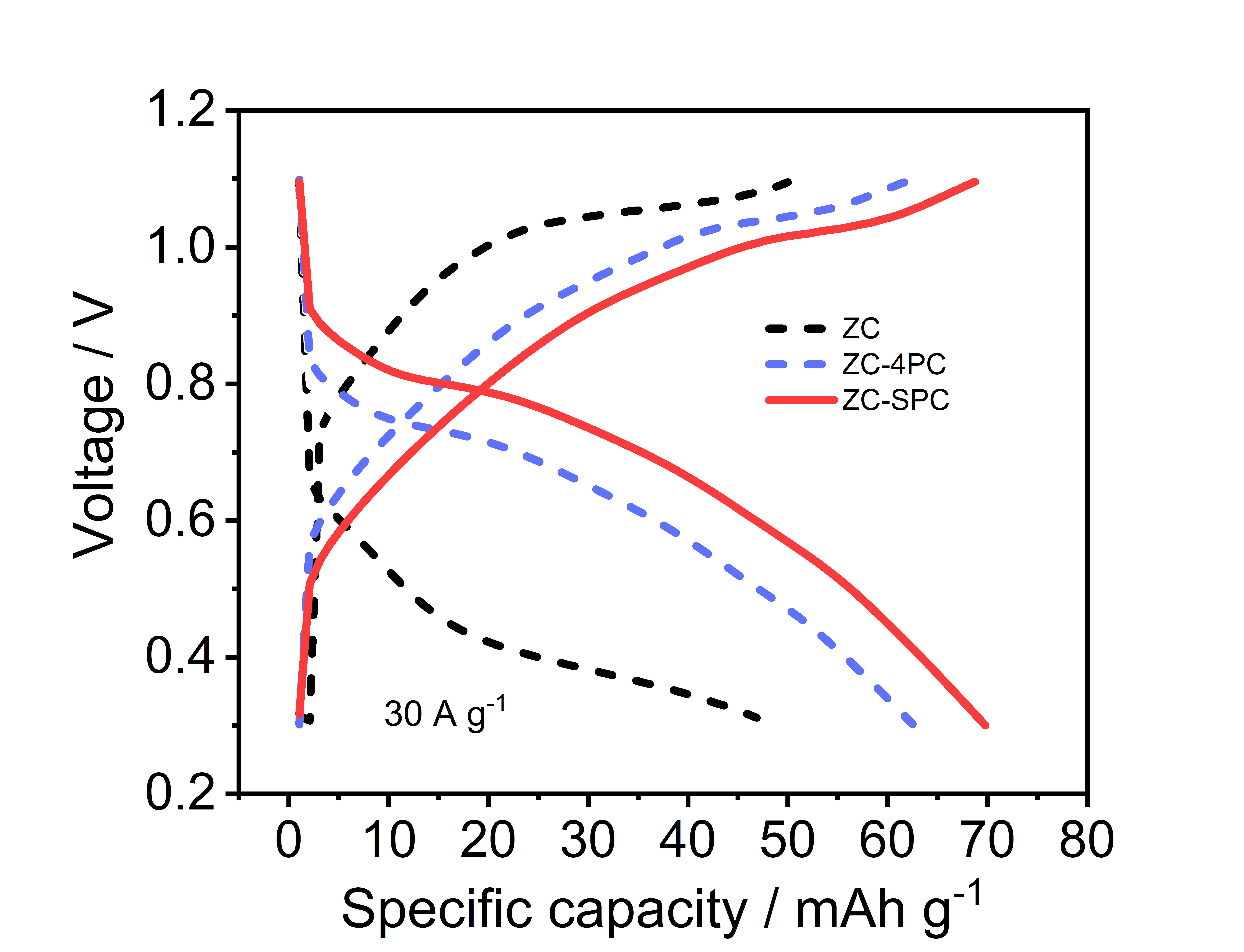


**Fig. S1** GCD profiles of Zn//PTCDA battery at 30 A g^−1^ within ZC, ZC-4PC, ZC-SPC electrolytes





**Fig. S2** 3D snapshot of ZC electrolyte obtained from MD simulations and partial enlarged snapshot representing Zn^2+^ inner solvation structure


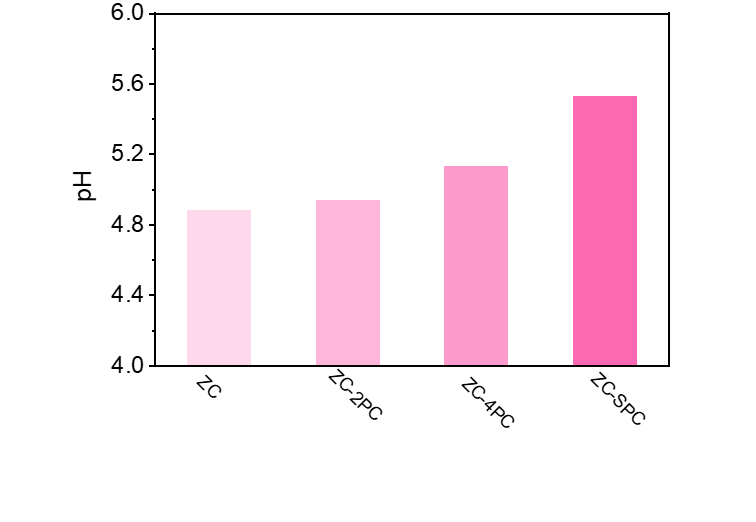


**Fig. S3** The pH of ZC, ZC-4PC, and ZC-SPC electrolytes


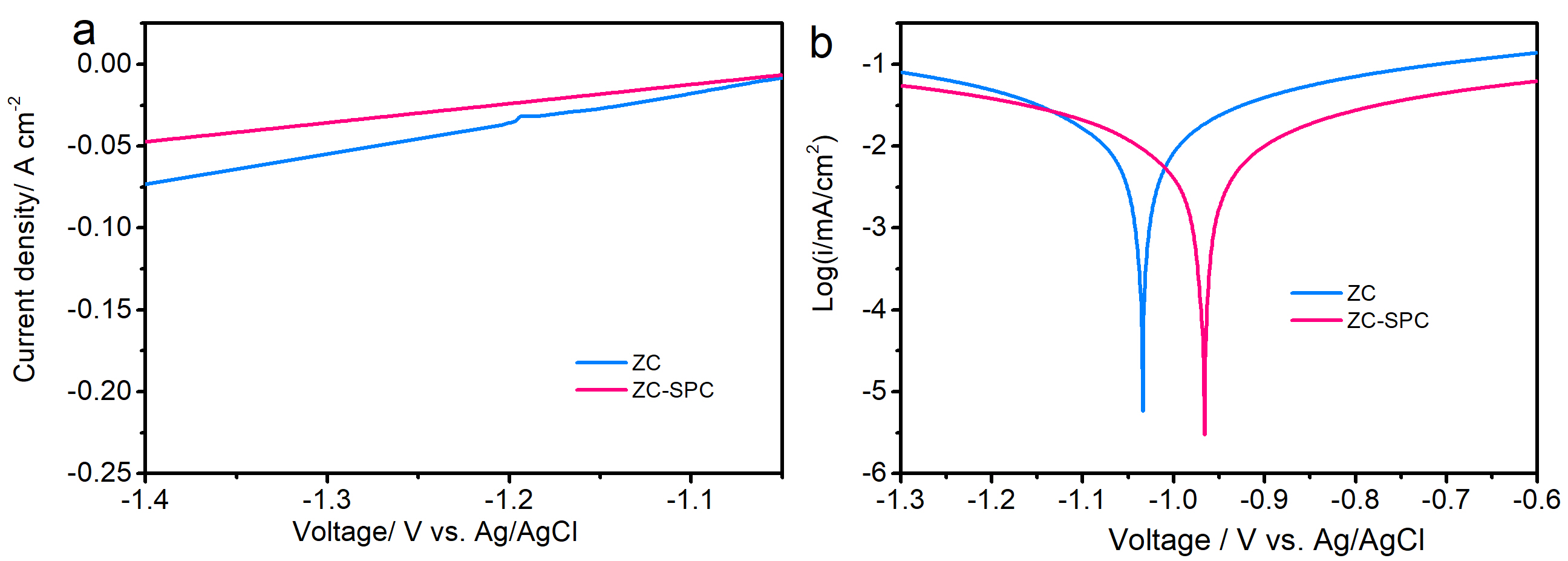


**Fig. S4** a) The LSV and b) Tafel plots of ZC and ZC‑SPC electrolytes, respectively.


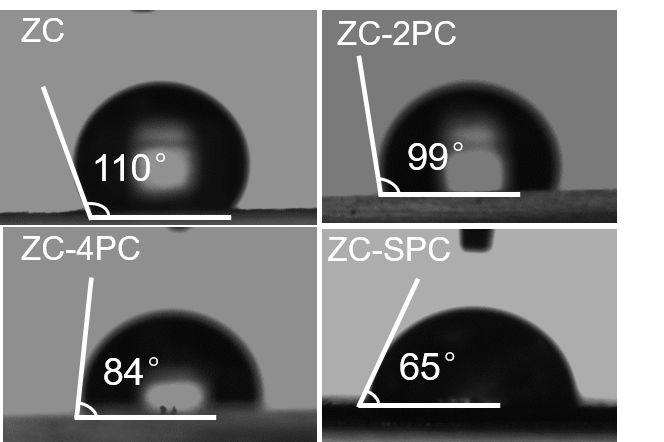


**Fig. S5** Contact angle of PTCDA electrode with ZC, ZC-2PC, ZC-4PC, and ZC-SPC electrolytes


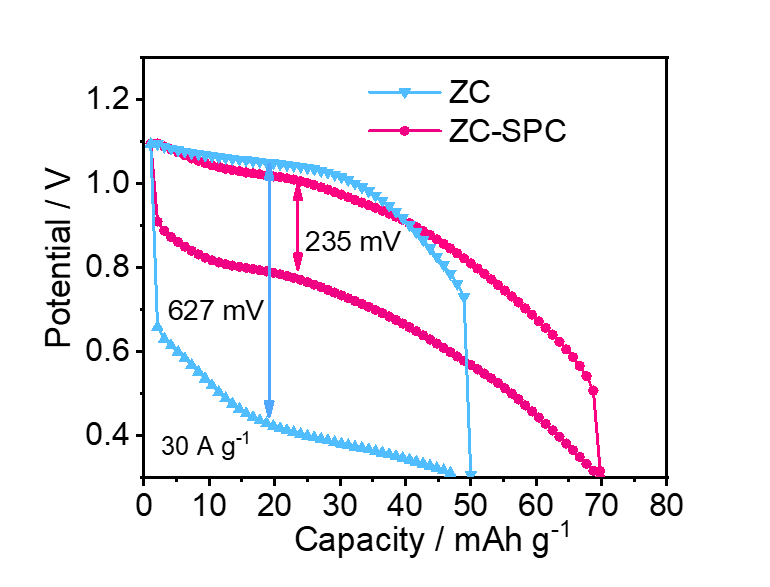


**Fig. S6** GCD profiles of Zn//PTCDA batteries at 30 A g^−1^


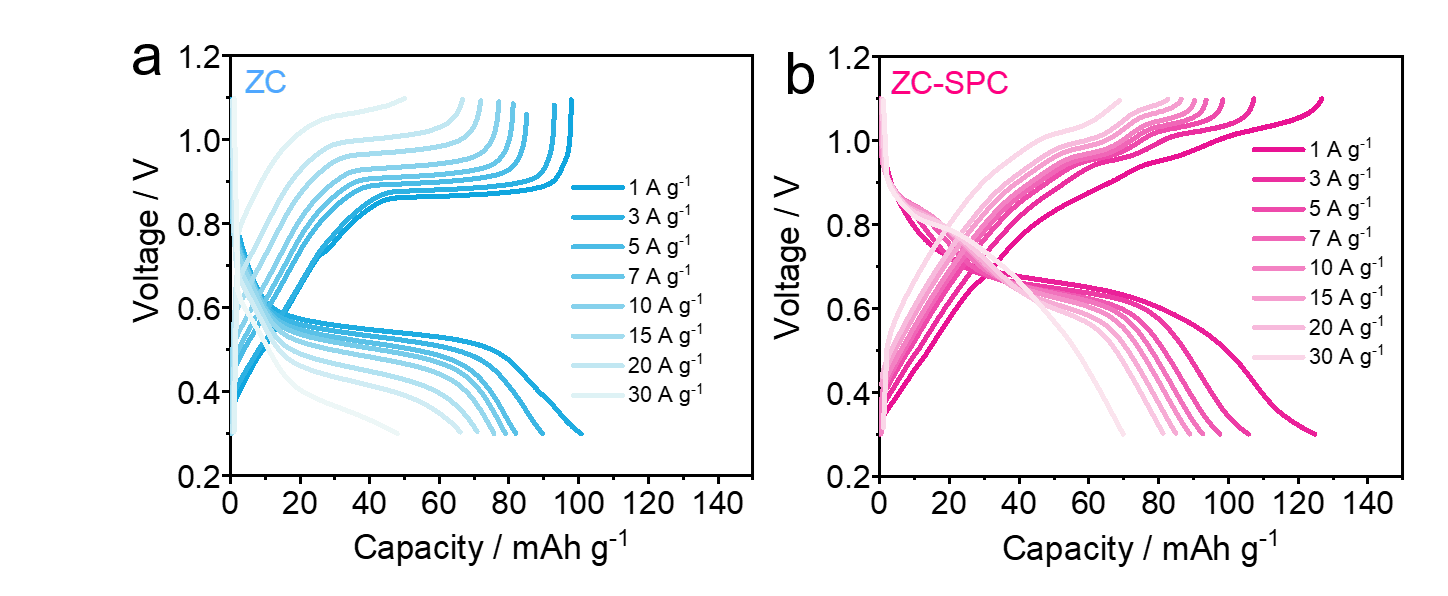


**Fig. S7** GCD profiles of Zn//PTCDA batteries at different current densities within a) ZC and b) ZC-SPC electrolytes


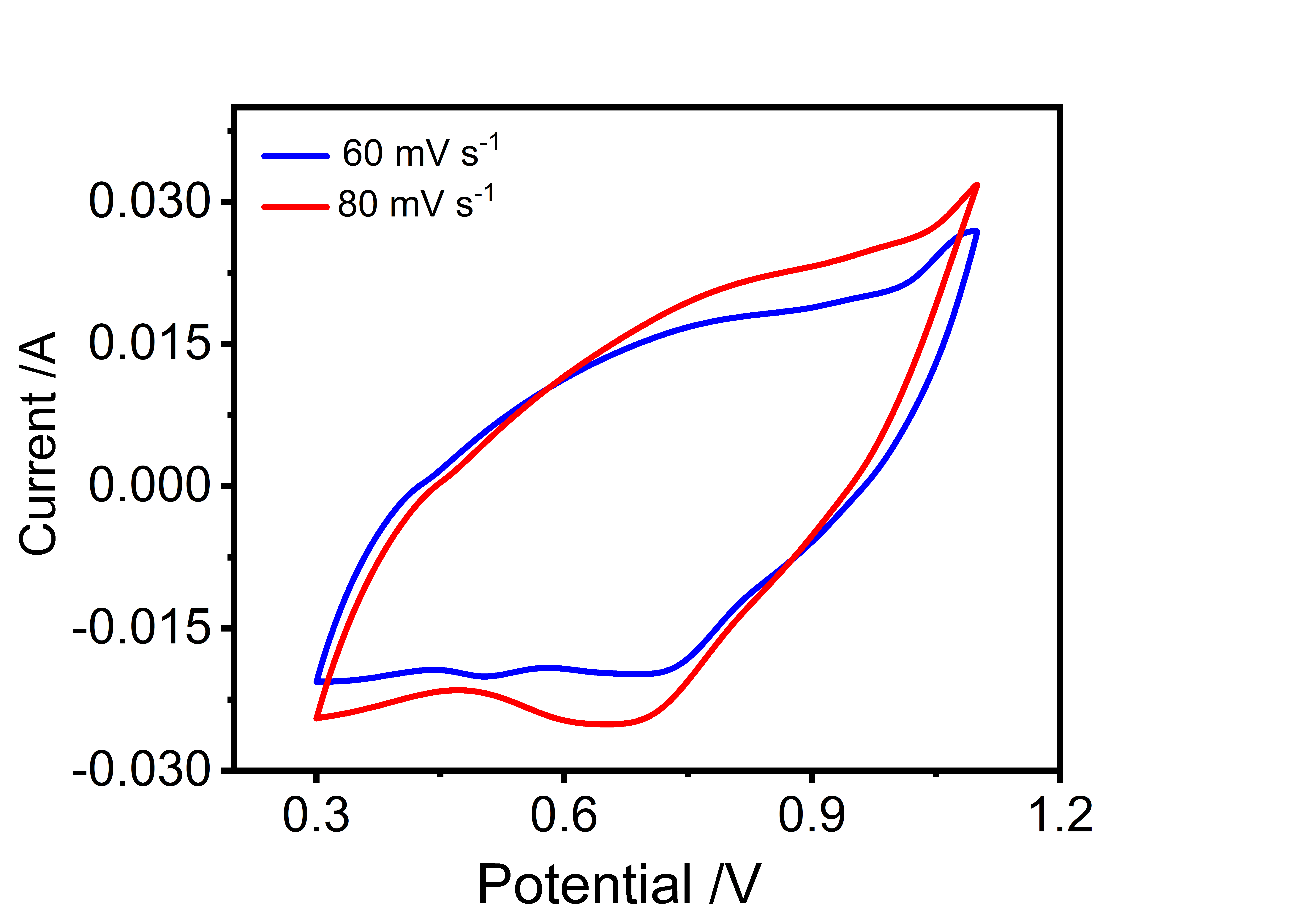


**Fig. S8** CV profiles of Zn//PTCDA batteries at current densities of 60 mV s^−1^ and 80 mV s^−1^ within ZC-SPC electrolytes


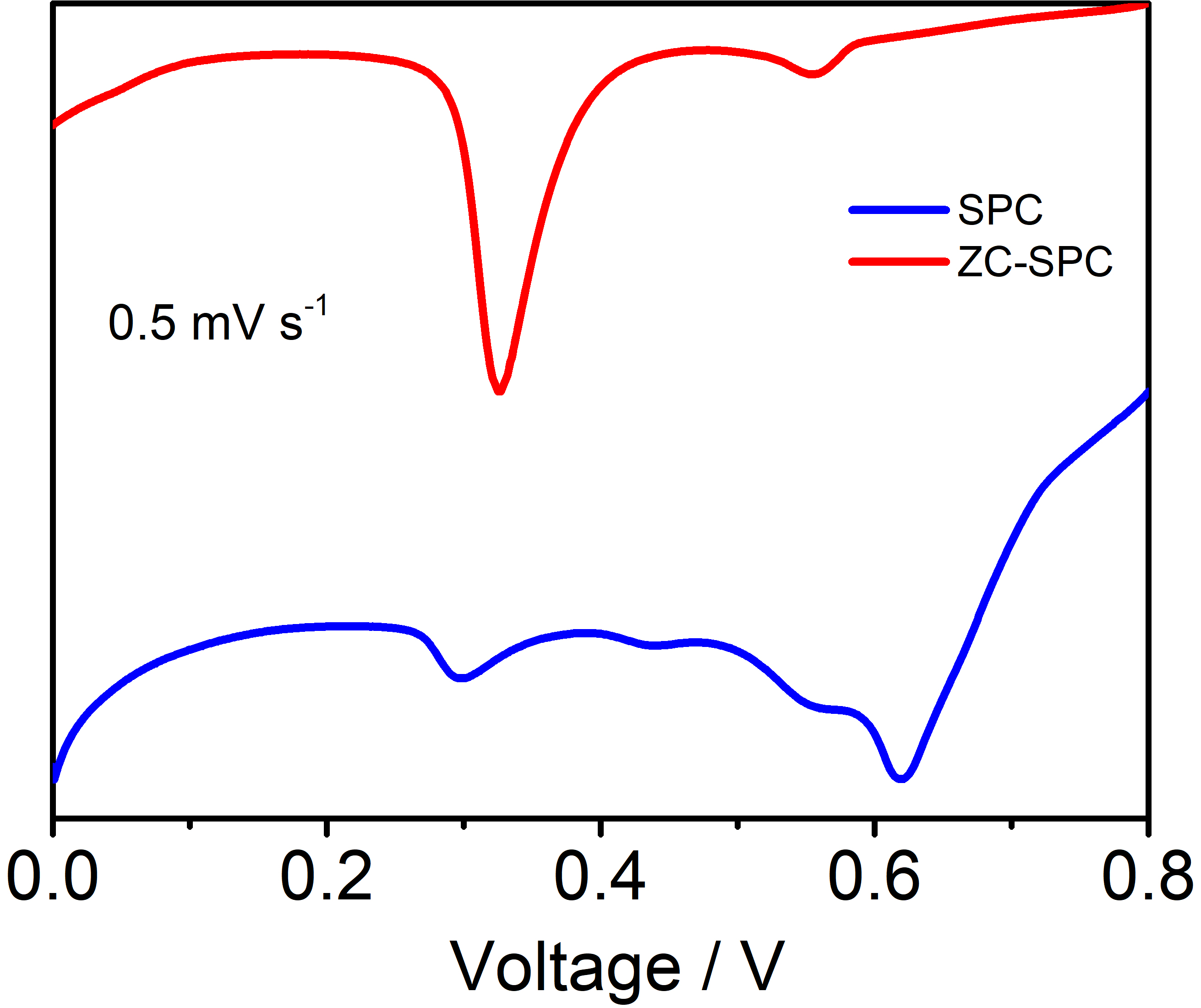


**Fig. S9** LSV profiles of Zn//PTCDA batteries measured at a scan rate of 0.5 mV s^−1^ in ZC-SPC and SPC electrolytes


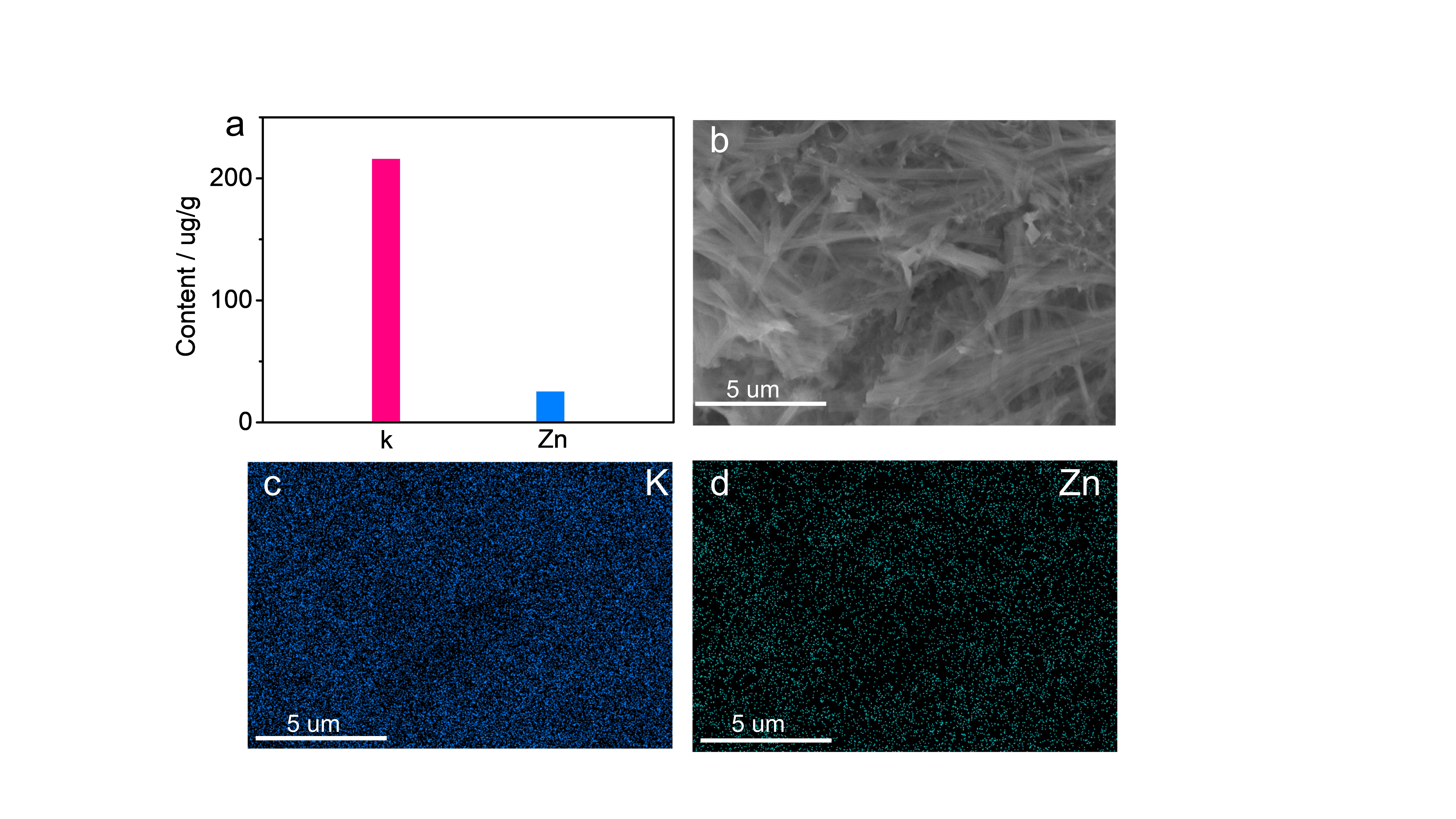


**Fig. S10** a) The measured K and Zn content in the PTCDA discharged state at 30 A g^−1^. b-d) SEM image and corresponding mapping of the PTCDA cathode in the discharged state at 30 A g^−1^


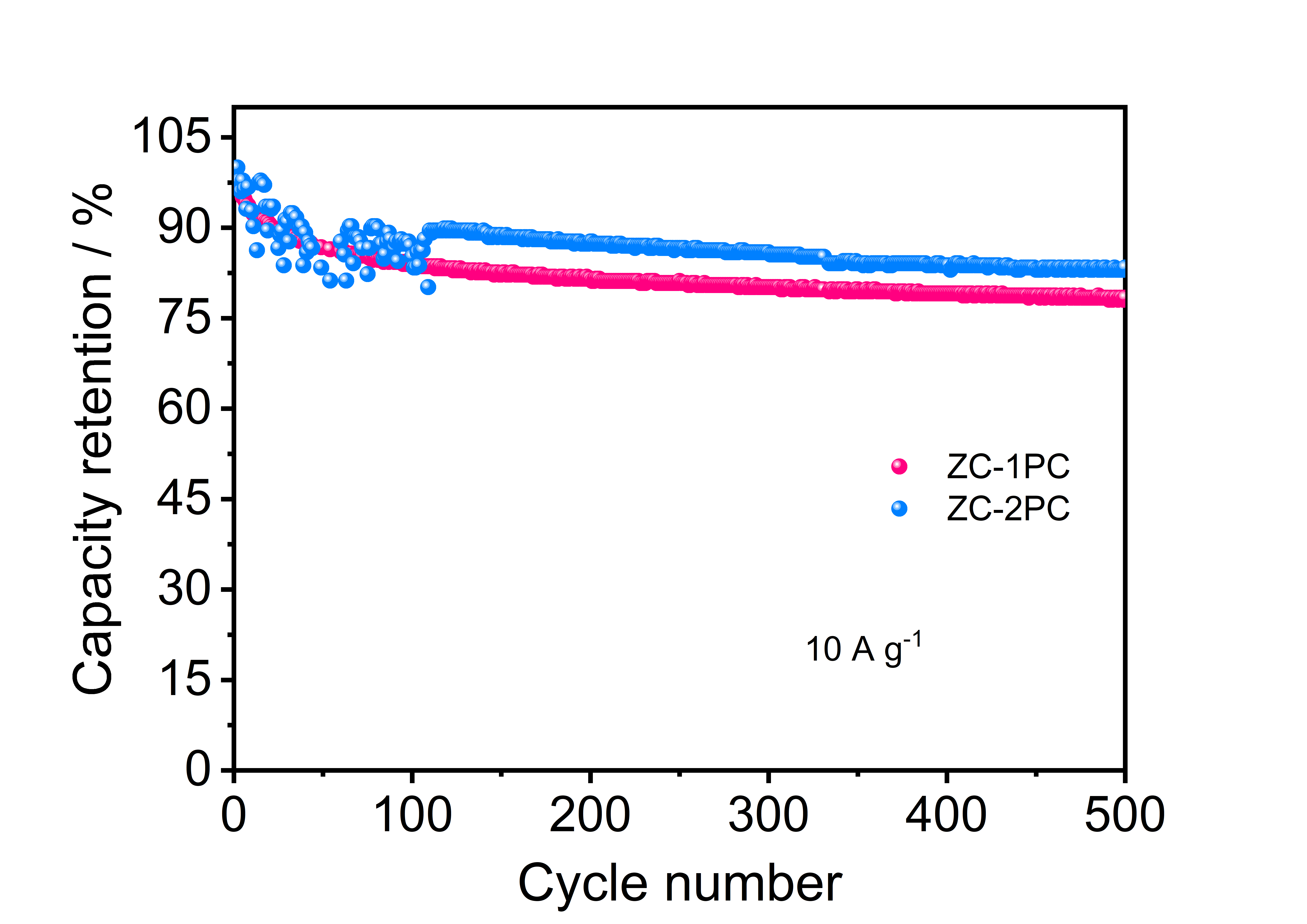


**Fig. S11** Cycling stability comparison of Zn//PTCDA batteries based on ZC-1PC and ZC-2PC electrolytes


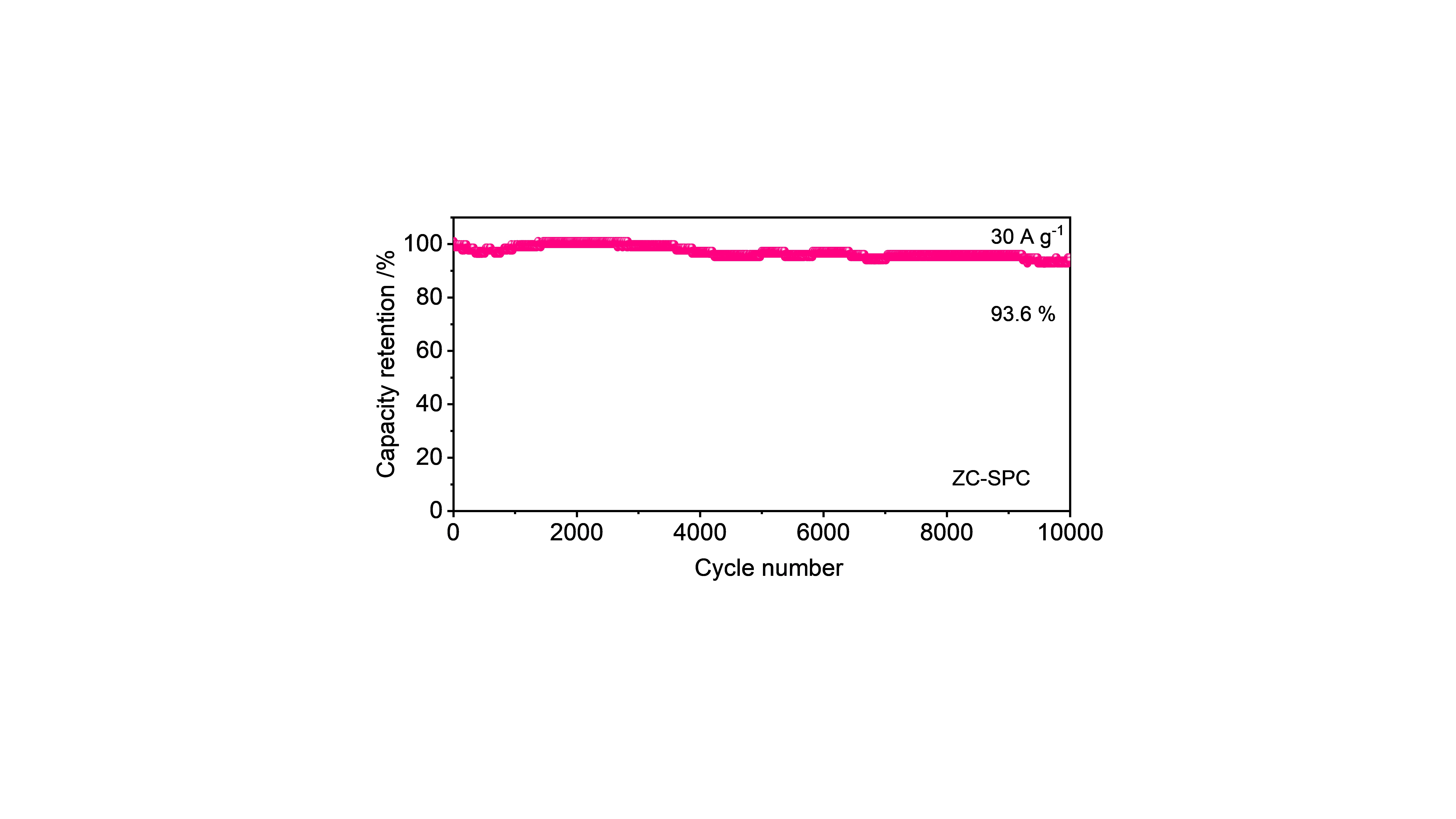


**Fig. S12** Cycling stability comparison of Zn//PTCDA batteries with ZC-SPC electrolyte at 30 A g^−1^


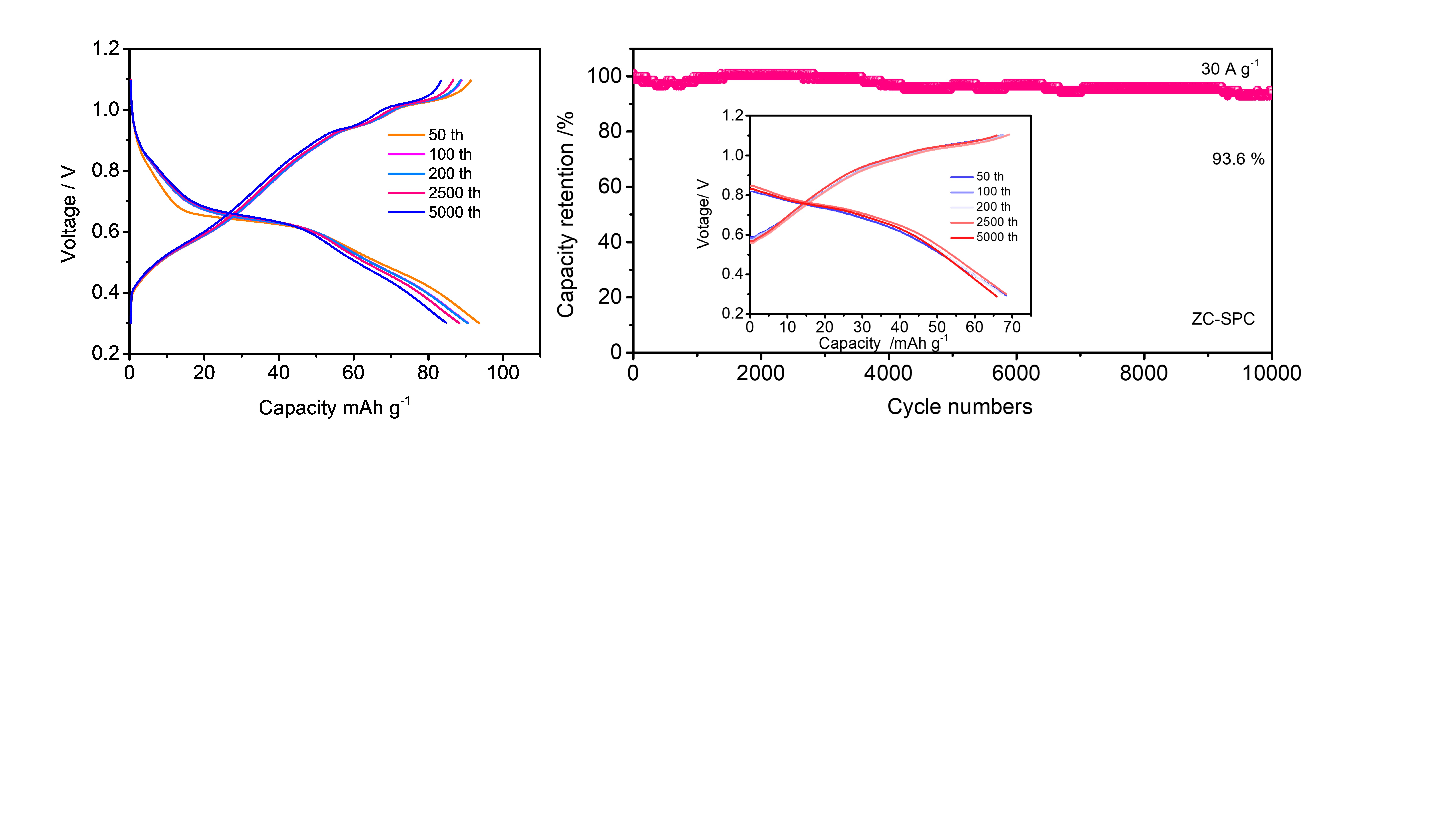


**Fig. S13** The GCD curves for Zn// PTCDA with ZC-SPC electrolyte from different cycles


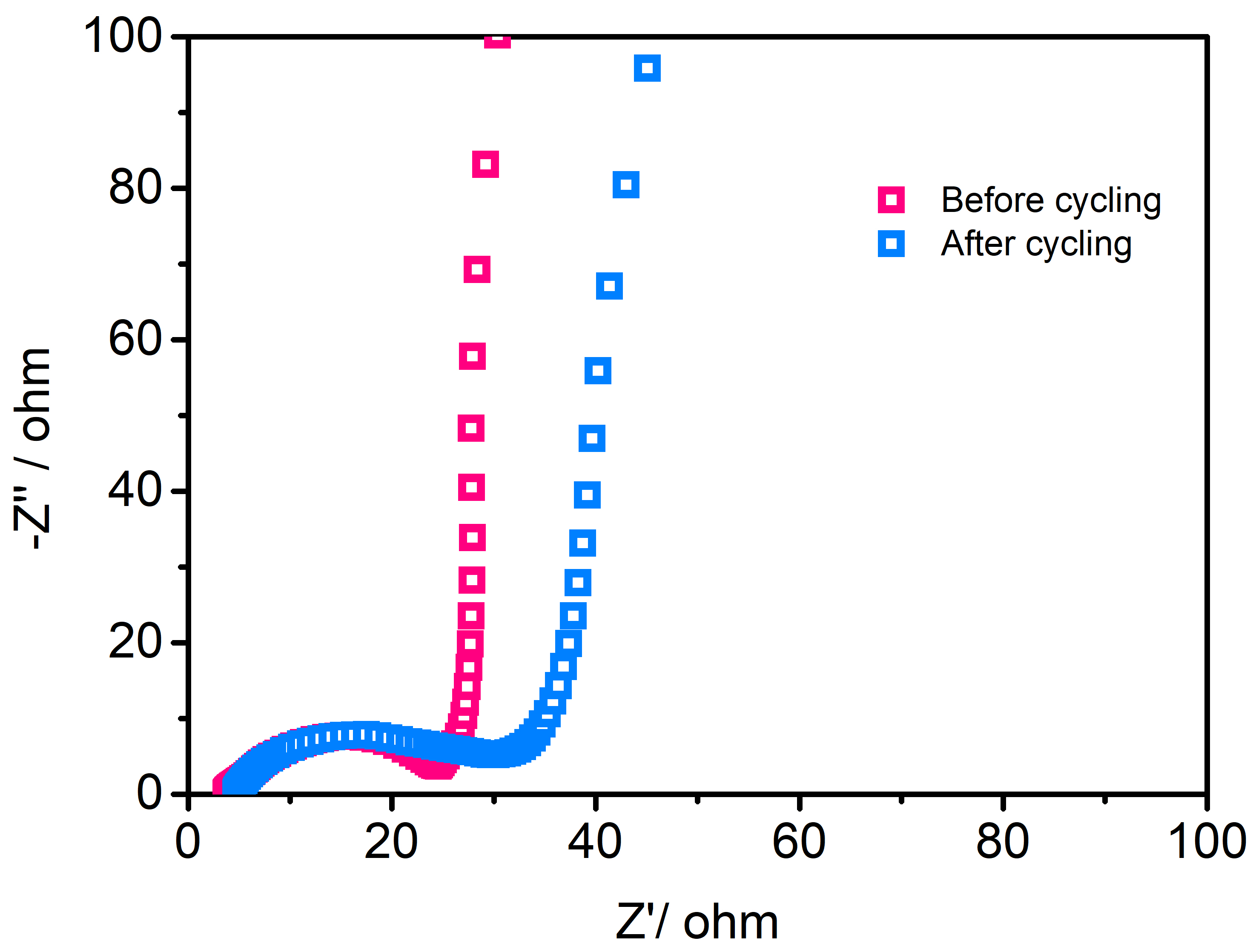


**Fig. S14** The EIS of Zn//PTCDA batteries before and after cycling


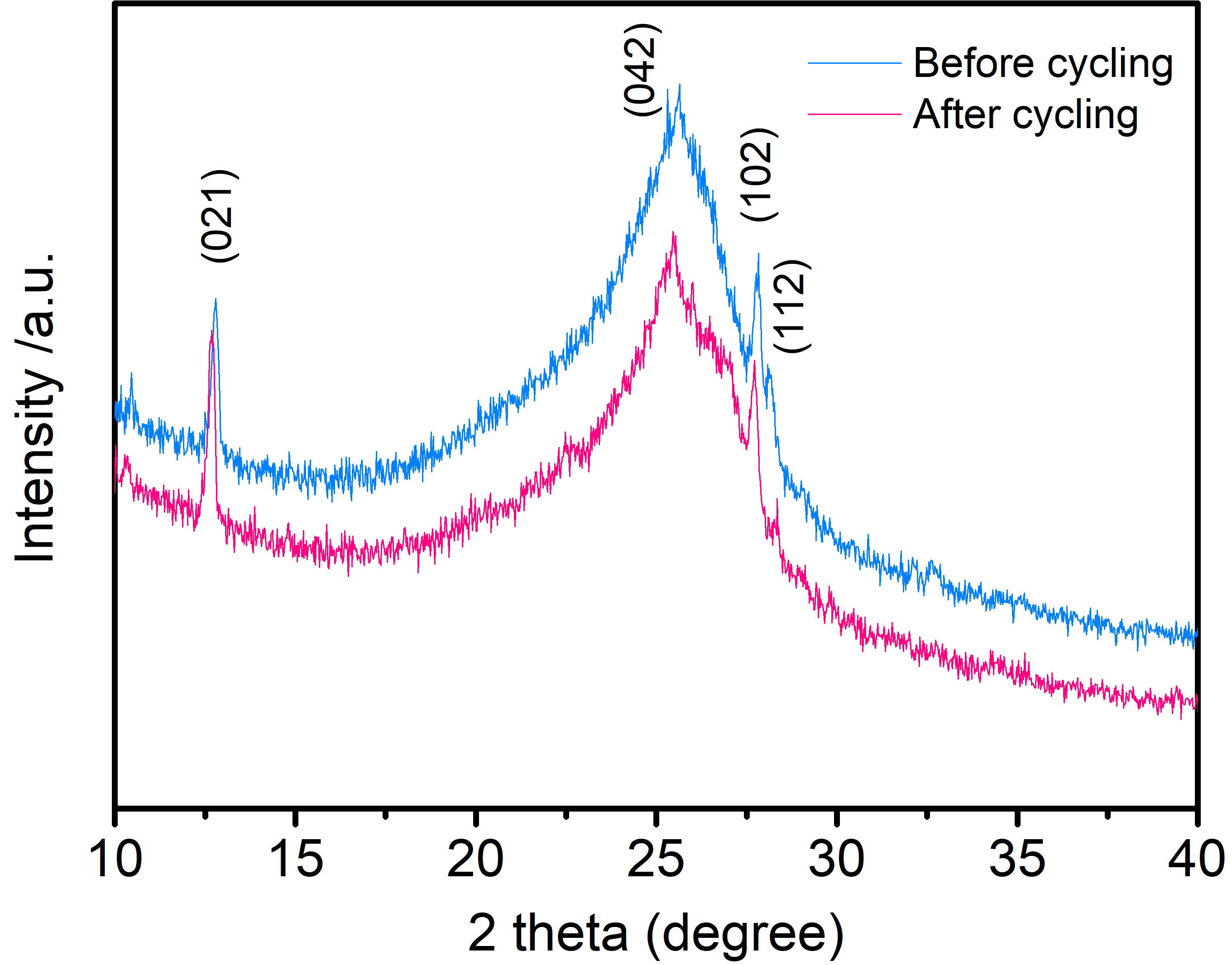


**Fig. S15** The XRD of PTCDA cathode for before and after cycling


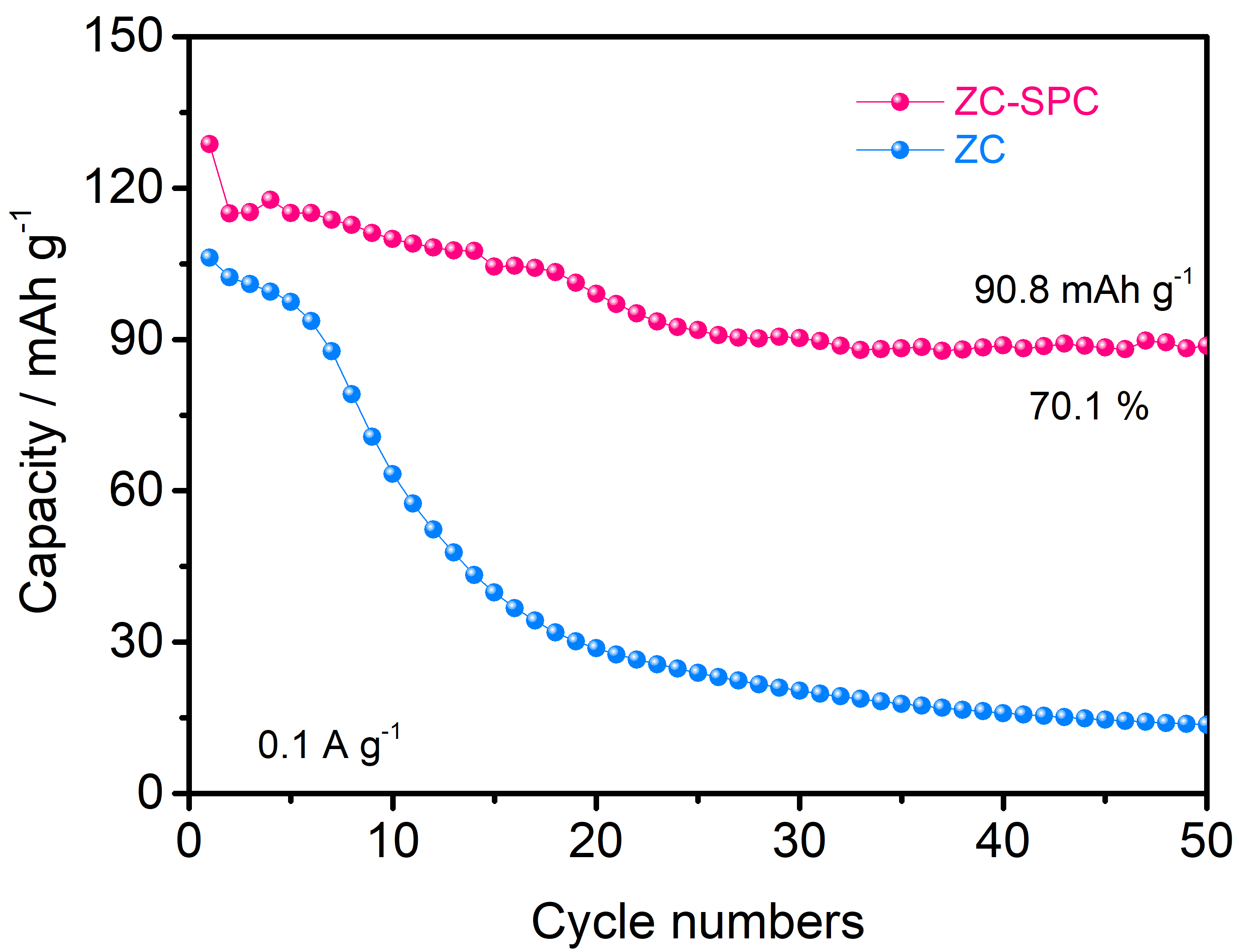


**Fig. S16** The cycling performance of Zn//PTCDA batteries at a current density of 0.1 A g^−1^ in the ZC and ZC‑SPC electrolytes


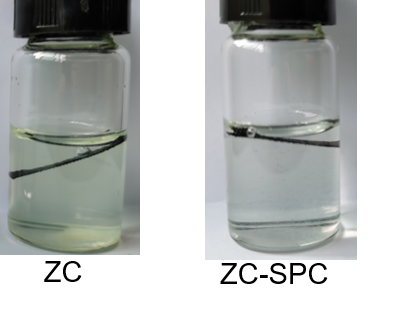


**Fig. S17** Optical images of ZC and ZC-SPC electrolytes after immersing PTCDA electrodes for 7 days


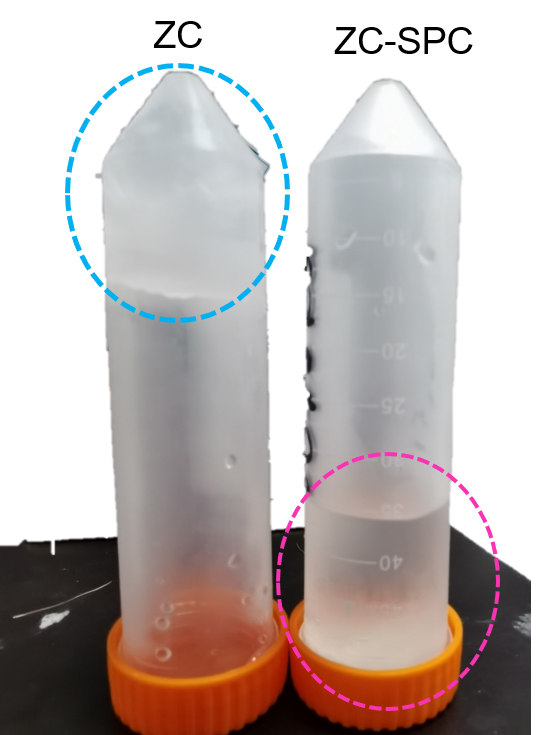


**Fig. S18** Optical images of ZC and ZC-SPC electrolytes after being stored at −20 °C for 72 hours


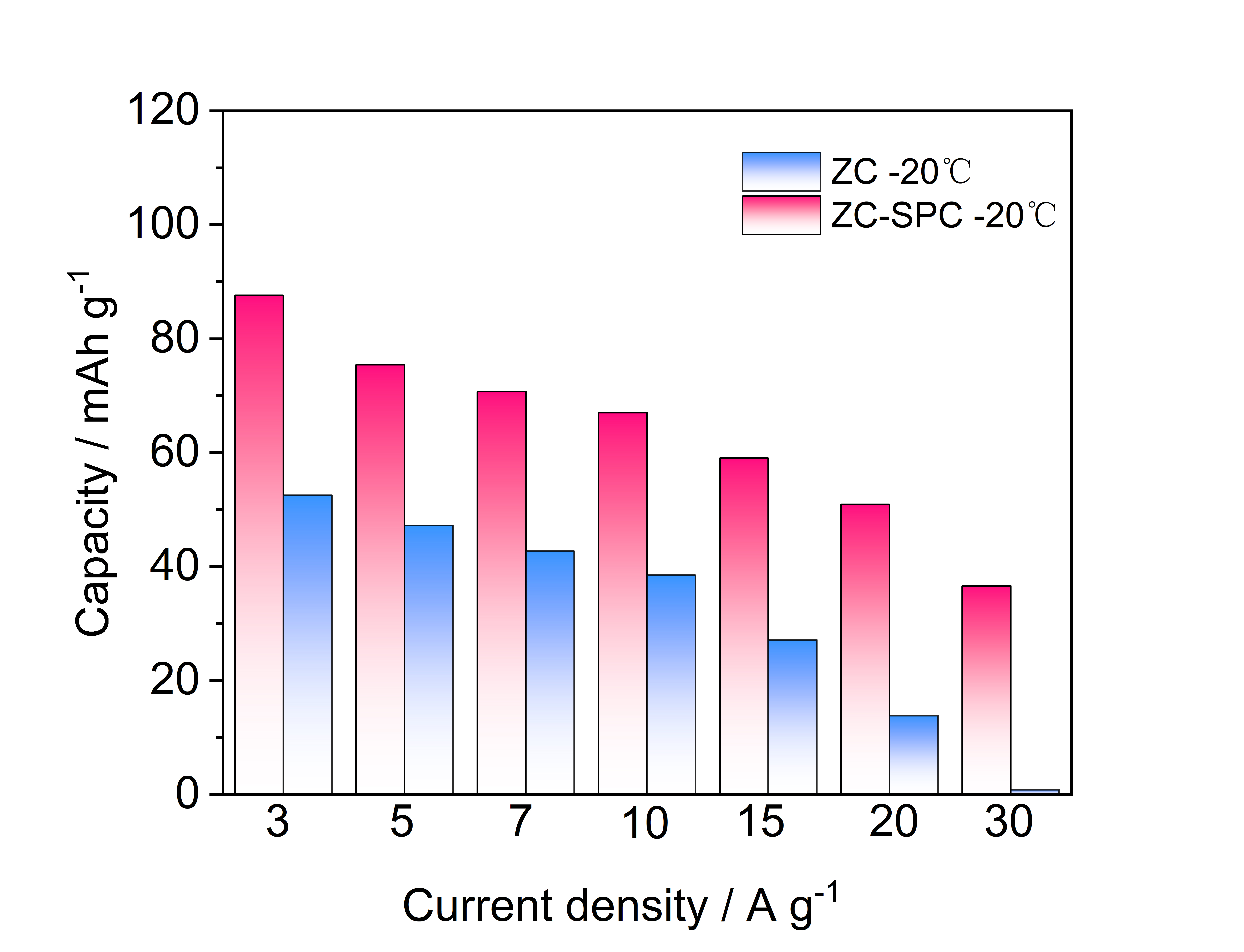


**Fig. S19** The rate performance of Zn//PTCDA batteries with the ZC and ZC-SPC electrolytes at −20 °C


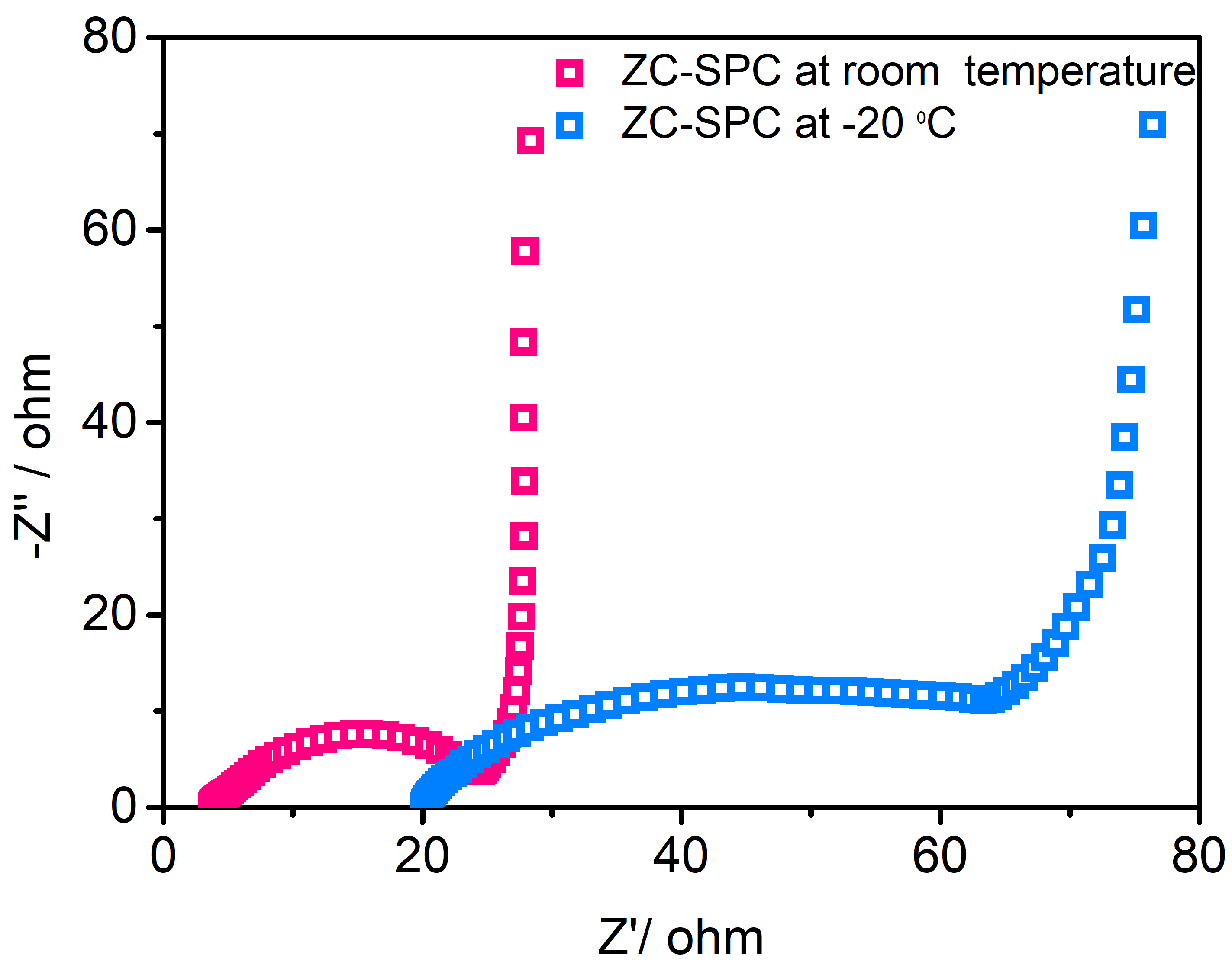


**Fig. S20** The EIS of ZC-SPC at room temperature and −20 ℃, respectively


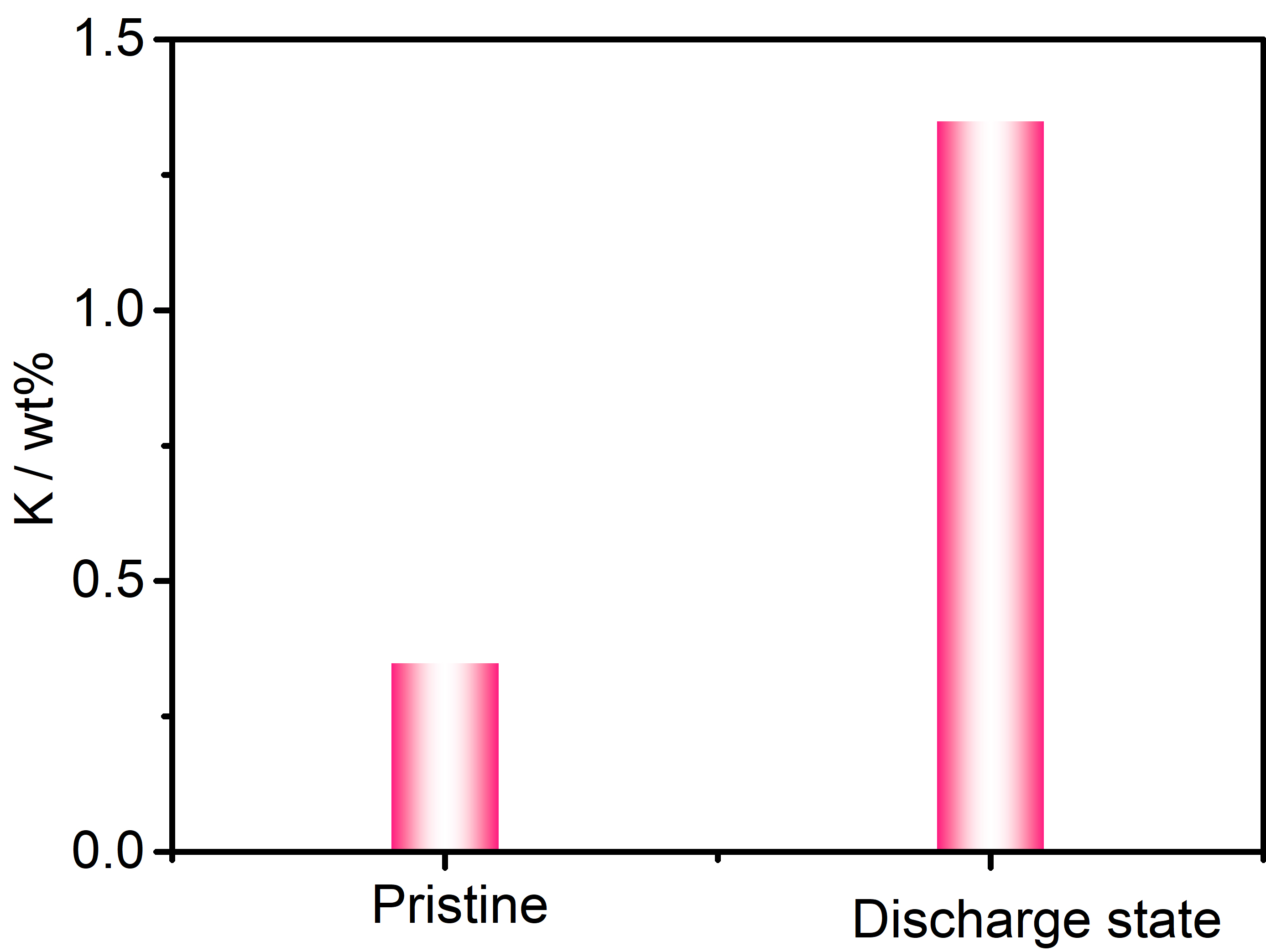


**Fig. S21** The measured K content in the PTCDA pristine and discharge state


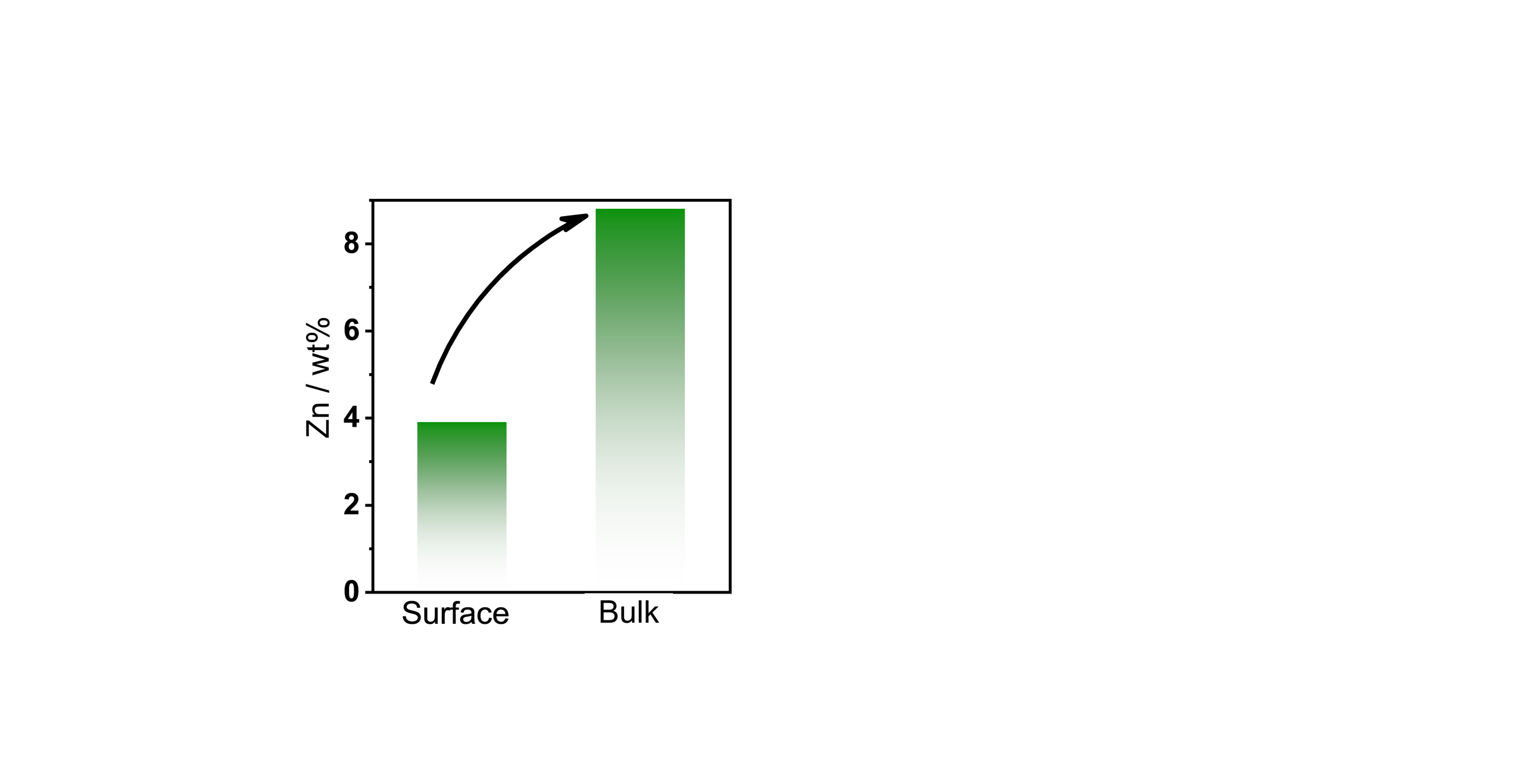


**Fig. S22** The content of Zn before and after sputtering


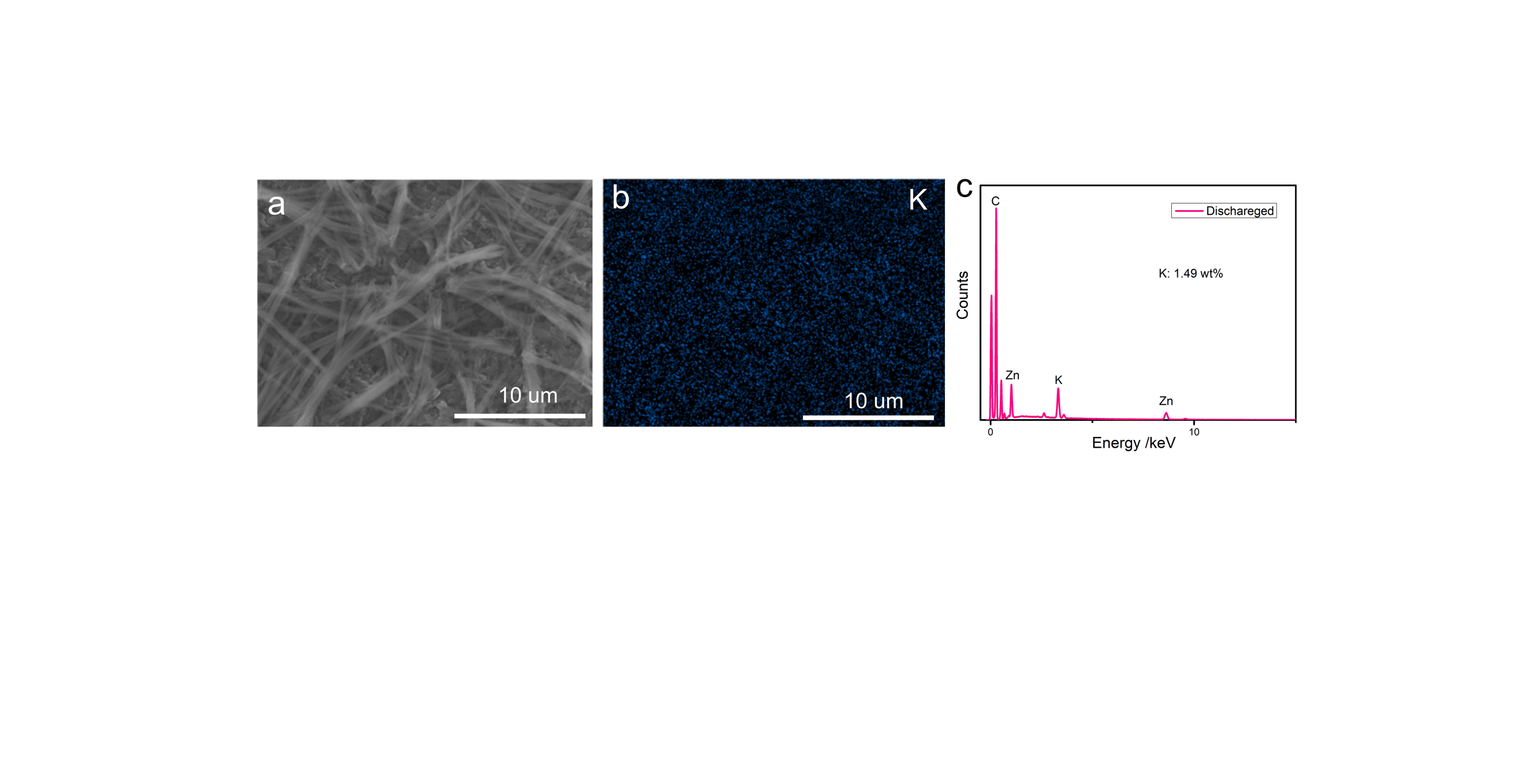


**Fig. S23** SEM image and corresponding EDS elemental mapping of the PTCDA cathode in the discharged state


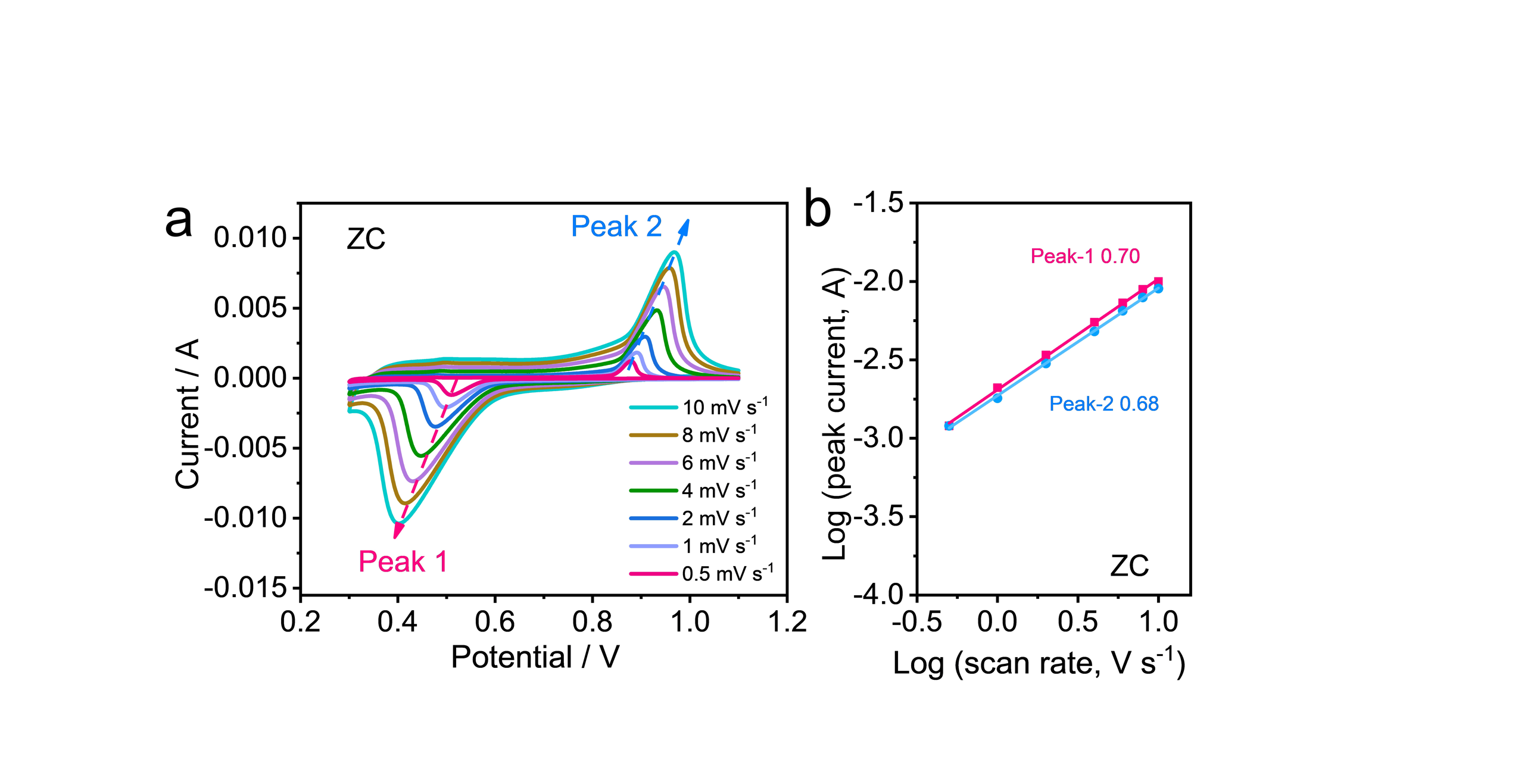


**Fig. S24** a) The CV curves of Zn//PTCDA batteries at different scan rates within ZC electrolytes. b) Log(*i*) versus log(*v*) plots of cathodic and anodic currents response at marked peaks in a)


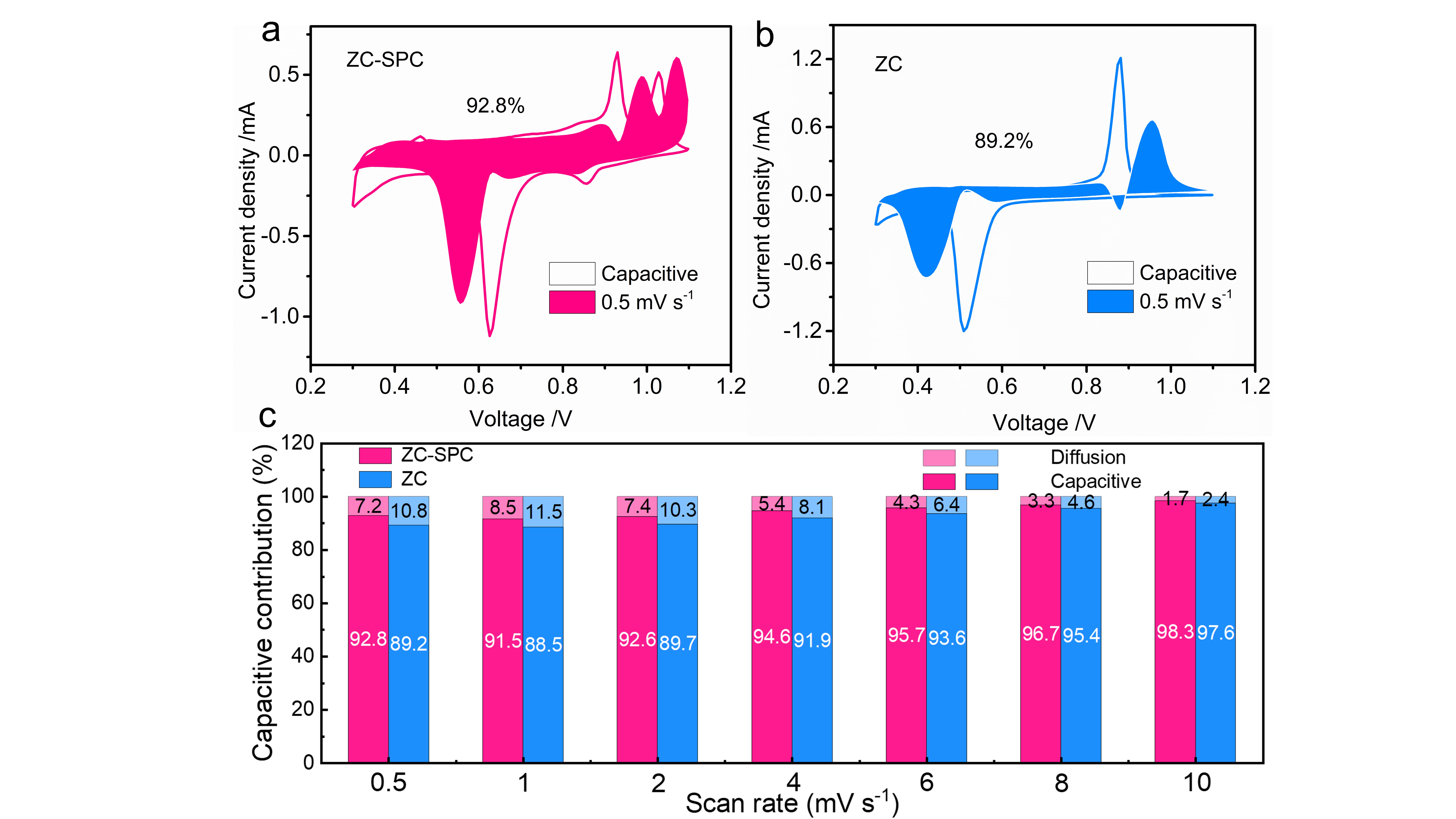


**Fig. S25** Contribution ratios of the capacitive-controlled (filled area) and diffusion-controlled (blank area) capacities of Zn//PTCDA for a) ZC-SPC and b) ZC electrolytes at a scan rate of 0.5 mV s^−1^. c) Bar diagram for contribution ratio between capacitive-controlled capacities and diffusion-controlled capacities at different scan rates


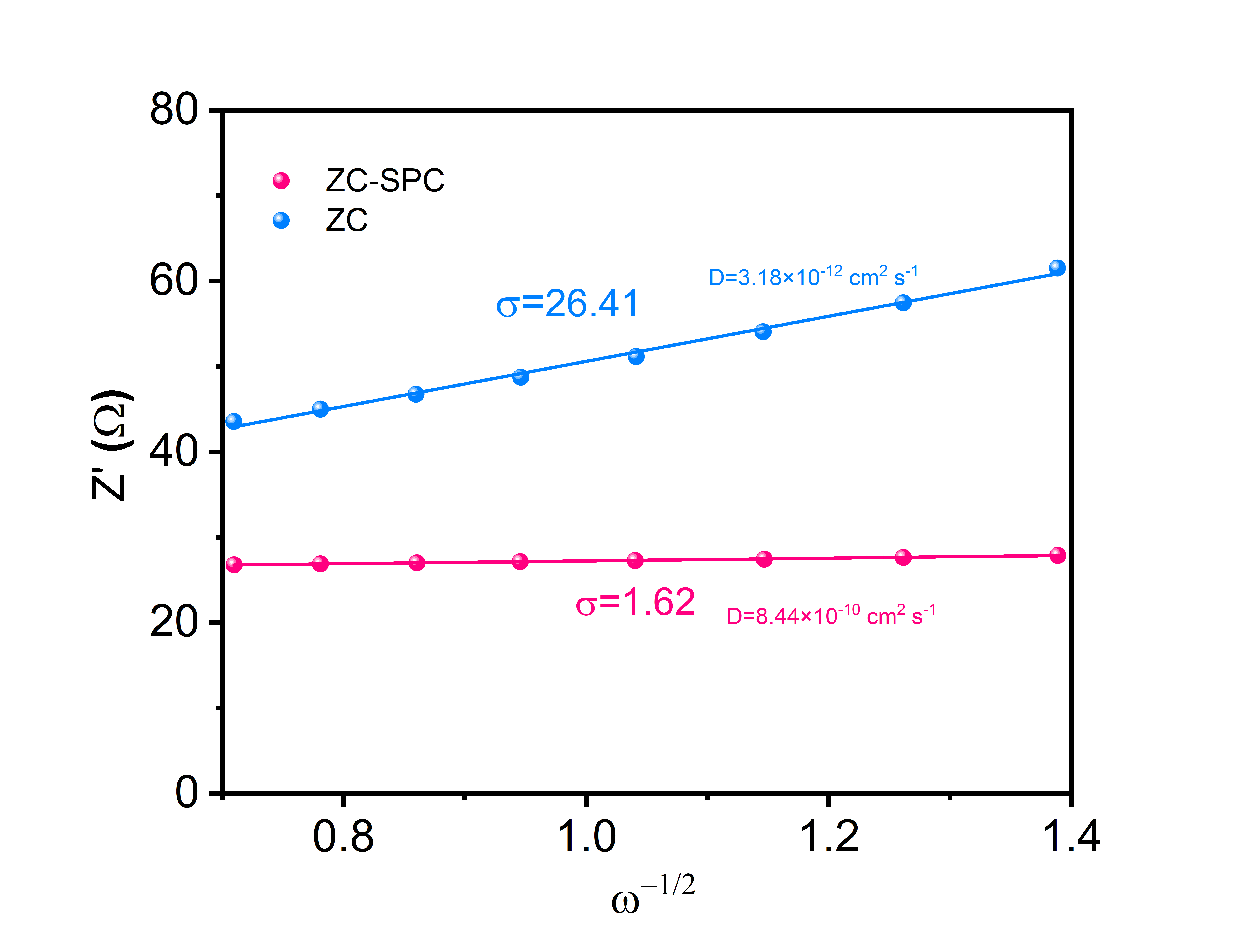


**Fig. S26** The calculate diffusion coefficients via EIS of Zn//PTCDA batteries with ZC and ZC-SPC electrolytes, respectively


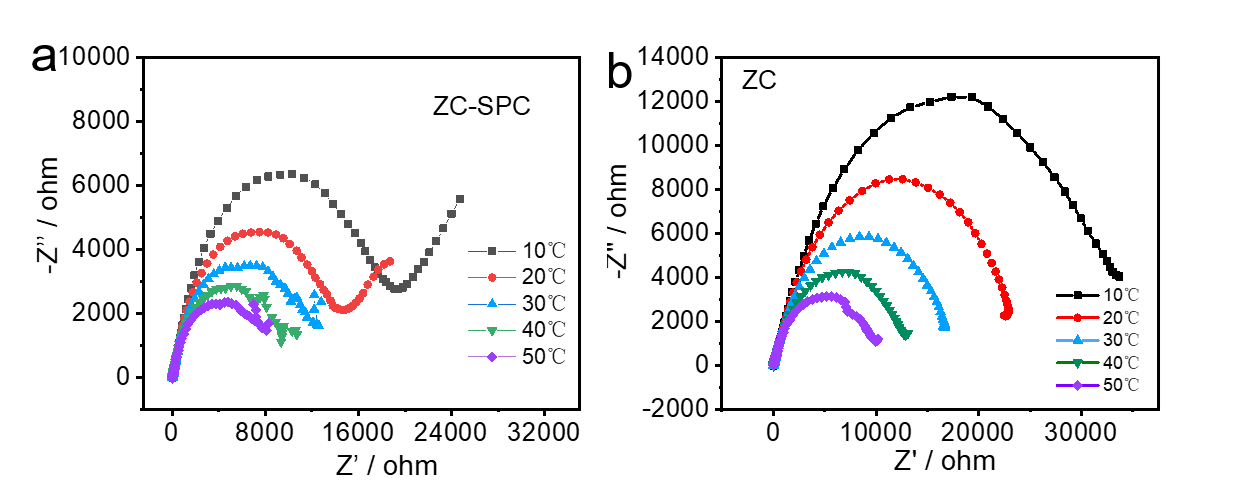


**Fig. S27** EIS curves tested under different temperature of (a) ZC-SPC and (b) ZC electrolytes


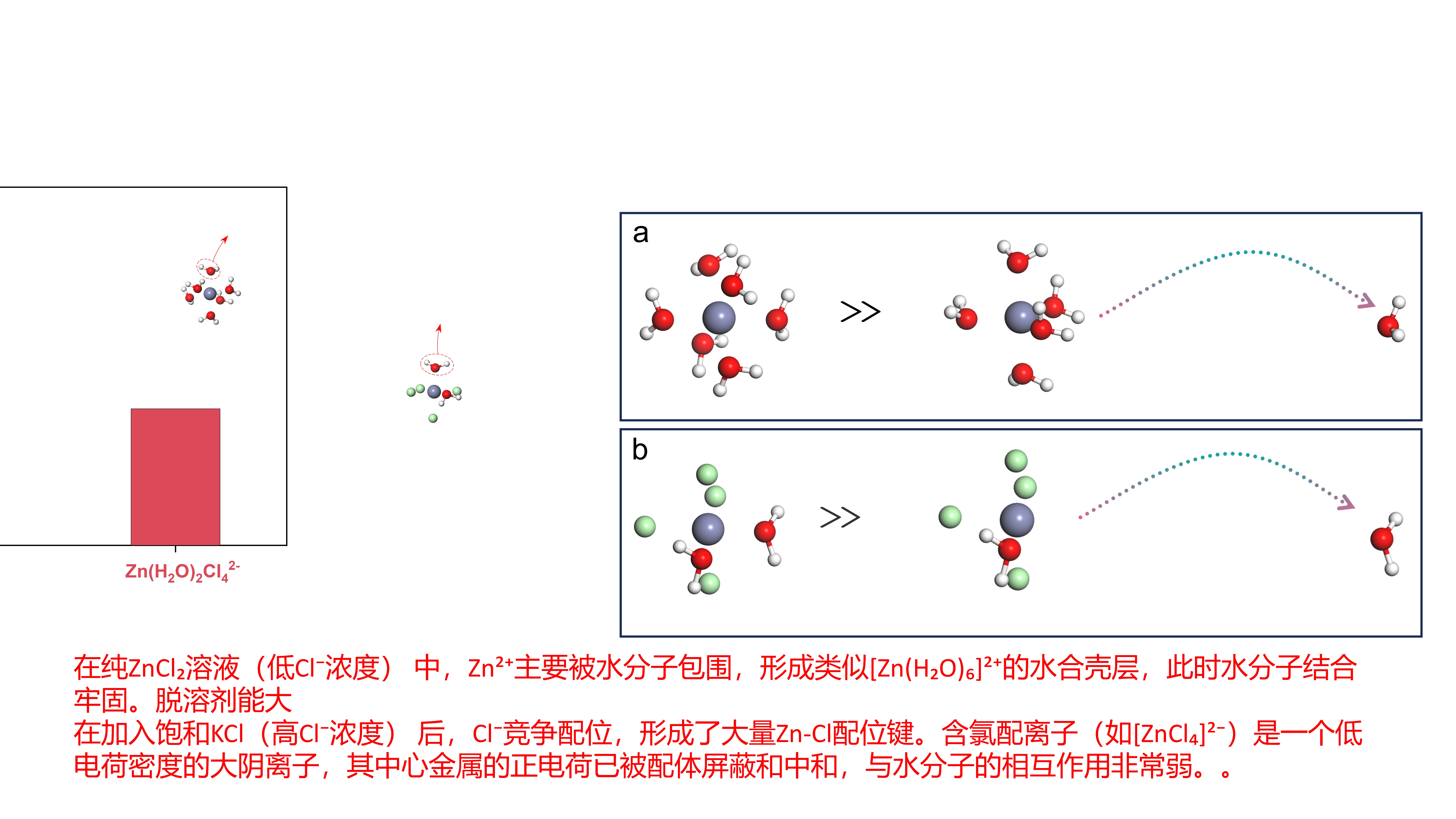


**Fig. S28** Schematic illustration of the desolvation process of (a) ZC and (b) ZC-SPC electrolytes





**Fig. S29** Schematic illustration of the intercalation process of (a) ZC-SPC and (b) ZC electrolytes

**S2 Supporting Table**

**Table S1** Comparison of electrochemical performance of organic materials

| Cathodes | | Electrolyte composition | | Capacity (mAh g^−1^) | Voltage (V) | | | | Rate capacity  (mAh g^−1^) | | Cycle life | Ref. |  |
| --- | --- | --- | --- | --- | --- | --- | --- | --- | --- | --- | --- | --- | --- |
| TRT | | 2 M ZnSO_4_ | 170 at 0.1 A g^−1^ | | | | 0.8 | | 100 at 2 A g^−1^ | 61.9%/1000 | | [S1] |  |
| TCNAQ | | 2 M ZnSO_4_ | 166 at 0.05 A g^−1^ | | | | 1.2 | | 55 at 1 A g^−1^ | 81%/1000 | | [S2] |  |
| PO | | 2 M ZnSO_4_ | 147 at 0.1 A g^−1^ | | | | 0.6 | | 90 at 1 A g^−1^ | 94%/500 | | [S3] |  |
| OAP | | 3 M Zn(CF_3_SO_3_)_2_ | 129.6 at 0.2 A g^−1^ | | | | 0.5 | | / | 85.8%/500 | | [S3] |  |
| PPy | PVA–KCl– Zn(CH_3_COO)_2_ gel | | 123 at 1.9 A g^−1^ | | | ~0.8 | | 60 at 21.5 A g^−1^ | | 38%/200 | | [S4] | |
| PDpBQ | | 2 M ZnSO_4_ | 120 at 0.1 A g^−1^ | | | | 1.16 | | 63 at 5 A g^−1^ | 79%/500 | | [S5] |  |
| PTDM | | 3 M Zn(OTF)_2_ | 118.3 at 0.1 A g^−1^ | | | | 1.13 | | 71.5 at 10 A g^−1^ | 65.6%/6400 | | [S6] |  |
| dNPC | | 20 m LiTFSI +1 m Zn(TFSI)_2_ | 109 at 0.05 A g^−1^ | | | | 1.3 | | 88 at 0.25 A g^−1^ | 96%/500 | | [S7] |  |
| PDA/CNT | | 3.3 M ZnSO_4_ | 85 at 1 A g^−1^ | | | | 0.9 | | 42 at 5 A g^−1^ | 96%/500 | | [S8] |  |
| π-PMC | | 2 M ZnCl_2_ | 122.9 at 0.2 A g^−1^ | | | | ~ 0.5 | | 76.9 at 32 A g^−1^ | 80.8%/500 | | [S9] |  |

**Supplementary References**

1. W. Wang, Y. Tang, J. Liu, H. Li, R. Wang et al., Boosting the zinc storage of a small-molecule organic cathode by a desalinization strategy. Chem. Sci. **14**, 9033-9040 (2023). https://doi.org/10.1039/D3SC03435F
2. Q. Wang, X. Xu, G. Yang, Y. Liu, X. Yao, An organic cathode with tailored working potential for aqueous Zn-ion batteries. Chem. Commun. **56**, 11859-11862 (2020). https://doi.org/10.1039/d0cc05344a
3. Z. Tie, S. Deng, H. Cao, M. Yao, Z. Niu et al., A symmetric all-organic proton battery in mild electrolyte. Angew. Chem. Int. Ed. **61**, 2115180 (2022). <https://doi.org/10.1002/anie.202115180>
4. J. Wang, J. Liu, M. Hu, J. Zeng, Y. Mu et al., A flexible, electrochromic, rechargeable Zn// PPy battery with a short circuit chromatic warning function. J. Mater. Chem. A **6**, 11113-11118 (2018). <https://doi.org/10.1039/c8ta03143f>
5. Wang, J. Xiao, W. Tang, Hydroquinone versus pyrocatechol pendants twisted conjugated polymer cathodes for high-performance and robust aqueous zinc-ion batteries. Adv. Funct. Mater. **32**, 2108225 (2022). <https://doi.org/10.1002/adfm.202108225>
6. Y. Wang, S. Qiu, D. He, J. Guo, M. Zhao et al., A high-potential bipolar phenothiazine derivative cathode for aqueous zinc batteries. ChemSusChem **16**, 2300658 (2023). https://doi.org/10.1002/cssc.202300658
7. U. Mittal, F. Colasuonno, A. Rawal, M. Lessio, D. Kundu, A highly stable 1.3V organic cathode for aqueous zinc batteries designed in situ by solid-state electrooxidation. Energy Storage Mater. **46**, 129-137 (2022). https://doi.org/10.1016/j.ensm.2022.01.004
8. X. Yue, H. Liu, P. Liu, Polymer grafted on carbon nanotubes as a flexible cathode for aqueous zinc ion batteries. Chem. Commun. **55**, 1647-1650 (2019). <https://doi.org/10.1039/C8CC10060H>
9. H. Zhang, Y. Fang, F. Yang, X. Liu, X. Lu, Aromatic organic molecular crystal with enhanced π–π stacking interaction for ultrafast zn-ion storage. Energy Environ. Sci. **13**(8), 2515-2523 (2020). https://doi.org/10.1039/D0EE01723J
